# miR-146a regulates insulin sensitivity via NPR3

**DOI:** 10.1007/s00018-020-03699-1

**Published:** 2020-11-18

**Authors:** Julian Roos, Meike Dahlhaus, Jan-Bernd Funcke, Monika Kustermann, Gudrun Strauss, Daniel Halbgebauer, Elena Boldrin, Karlheinz Holzmann, Peter Möller, Bernadette M. Trojanowski, Bernd Baumann, Klaus-Michael Debatin, Martin Wabitsch, Pamela Fischer-Posovszky

**Affiliations:** 1 Department of Pediatrics and Adolescent Medicine, University Medical Center, Eythstr. 24, 89075 Ulm, Germany; 2 Division of Pediatric Endocrinology and Diabetes, Department of Pediatrics and Adolescent Medicine, University Medical Center, Ulm, Germany; 3 Department of Internal Medicine, Touchstone Diabetes Center, The University of Texas Southwestern Medical Center, Dallas, TX USA; 4 Core Facility Genomics, Ulm University, Ulm, Germany; 5 Institute of Pathology, University Medical Center, Ulm, Germany; 6 Institute of Physiological Chemistry, Ulm University, Ulm, Germany

**Keywords:** microRNA, Adipocyte, Insulin resistance, NPR3

## Abstract

The pathogenesis of obesity-related metabolic diseases has been linked to the inflammation of white adipose tissue (WAT), but the molecular interconnections are still not fully understood. MiR-146a controls inflammatory processes by suppressing pro-inflammatory signaling pathways. The aim of this study was to characterize the role of miR-146a in obesity and insulin resistance. MiR-146a^−/−^ mice were subjected to a high-fat diet followed by metabolic tests and WAT transcriptomics. Gain- and loss-of-function studies were performed using human Simpson–Golabi–Behmel syndrome (SGBS) adipocytes. Compared to controls, miR-146a^−/−^ mice gained significantly more body weight on a high-fat diet with increased fat mass and adipocyte hypertrophy. This was accompanied by exacerbated liver steatosis, insulin resistance, and glucose intolerance. Likewise, adipocytes transfected with an inhibitor of miR-146a displayed a decrease in insulin-stimulated glucose uptake, while transfecting miR-146a mimics caused the opposite effect. Natriuretic peptide receptor 3 (NPR3) was identified as a direct target gene of miR-146a in adipocytes and CRISPR/Cas9-mediated knockout of NPR3 increased insulin-stimulated glucose uptake and enhanced de novo lipogenesis. In summary, miR-146a regulates systemic and adipocyte insulin sensitivity via downregulation of NPR3.

**Electronic supplementary material:**

The online version of this article (10.1007/s00018-020-03699-1) contains supplementary material, which is available to authorized users.

## Introduction

Obesity is associated with white adipose tissue (WAT) inflammation and insulin resistance [1]. It is well documented that cells of the innate as well as the adaptive immune system accumulate in expanding WAT and by virtue of their secretion products, inhibit the action of insulin in adipocytes [1]. Consequently, insulin-resistant and dysfunctional WAT is no longer capable of expanding appropriately to store excess energy in a safe manner, causing ectopic lipid accumulation and progressive systemic insulin resistance [2].

MicroRNAs (miRNA) are highly conserved, non-coding regulatory RNAs of 19–24 nucleotides length. They affect the translation and stability of messenger RNAs (mRNAs) through RNA-RNA interactions and thereby regulate a plethora of biological processes. MiRNAs are also important mediators of adipose tissue function. They play a role in white and brown adipocyte differentiation [3], regulate mitochondrial function [4], and modulate insulin sensitivity [5]. Our group recently identified 24 miRNAs that were differentially regulated in a human in vitro model of adipose tissue inflammation, among them miR-146a, which was robustly induced in adipocytes under inflammatory conditions [6]. Likewise, miR-146a was highly expressed in both murine and human obese adipose tissue and we identified its function as a fine tuner of the adipocyte inflammatory response [6]. MiR-146a is a well-studied miRNA in the fields of cancer and immunology and is known for its anti-inflammatory effects in different cells types [7]. Runtsch et al*.* recently reported that miR-146a^−/−^ mice exhibit increased weight gain and adiposity and a disturbed insulin sensitivity when fed a high-fat diet (HFD) [8]. An increased inflammation in WAT and especially the dysregulation of metabolic pathways within infiltrating macrophages were presented as mode of action of miR-146a in obese WAT. Whether miR-146a regulates insulin sensitivity at the level of insulin-responsive cells, independently of inflammatory processes, has however not been addressed so far.

In this study, we hypothesized that miR-146a serves as a regulator of insulin sensitivity in adipocytes. To this end, miR-146a^−/−^ mice were subjected to a HFD followed by metabolic tests and WAT transcriptomics. Gain- and loss-of-function studies in human Simpson–Golabi–Behmel syndrome (SGBS) adipocytes resulted in the identification of natriuretic peptide receptor 3 (NPR3) as target gene of miR-146a and as regulator of adipocyte insulin sensitivity.

## Results

### Basal characterization

To study the impact of miR-146a on insulin sensitivity, we took advantage of a knockout model originally described by Boldin et al*.* and Lu et al*.* [9, 10]. At an age of 10 weeks, miR-146a^−/−^ mice (KO) had a slightly, but significantly higher body weight compared to control mice (WT) (Supplemental Fig. S1A). Fasting glucose and insulin levels were not different, resulting in a similar HOMA-IR index (Supplemental Fig. S1B–D). In an intraperitoneal insulin tolerance test (ITT), miR-146a^−/−^ mice exhibited slightly impaired insulin sensitivity (Supplemental Fig. S1E), yet comparable glucose tolerance as measured in an oral glucose tolerance test (OGTT) (Supplemental Fig. S1F).

### Increased body weight and fat mass in miR-146a^−/−^ mice upon HFD feeding

**Fig. 1:**
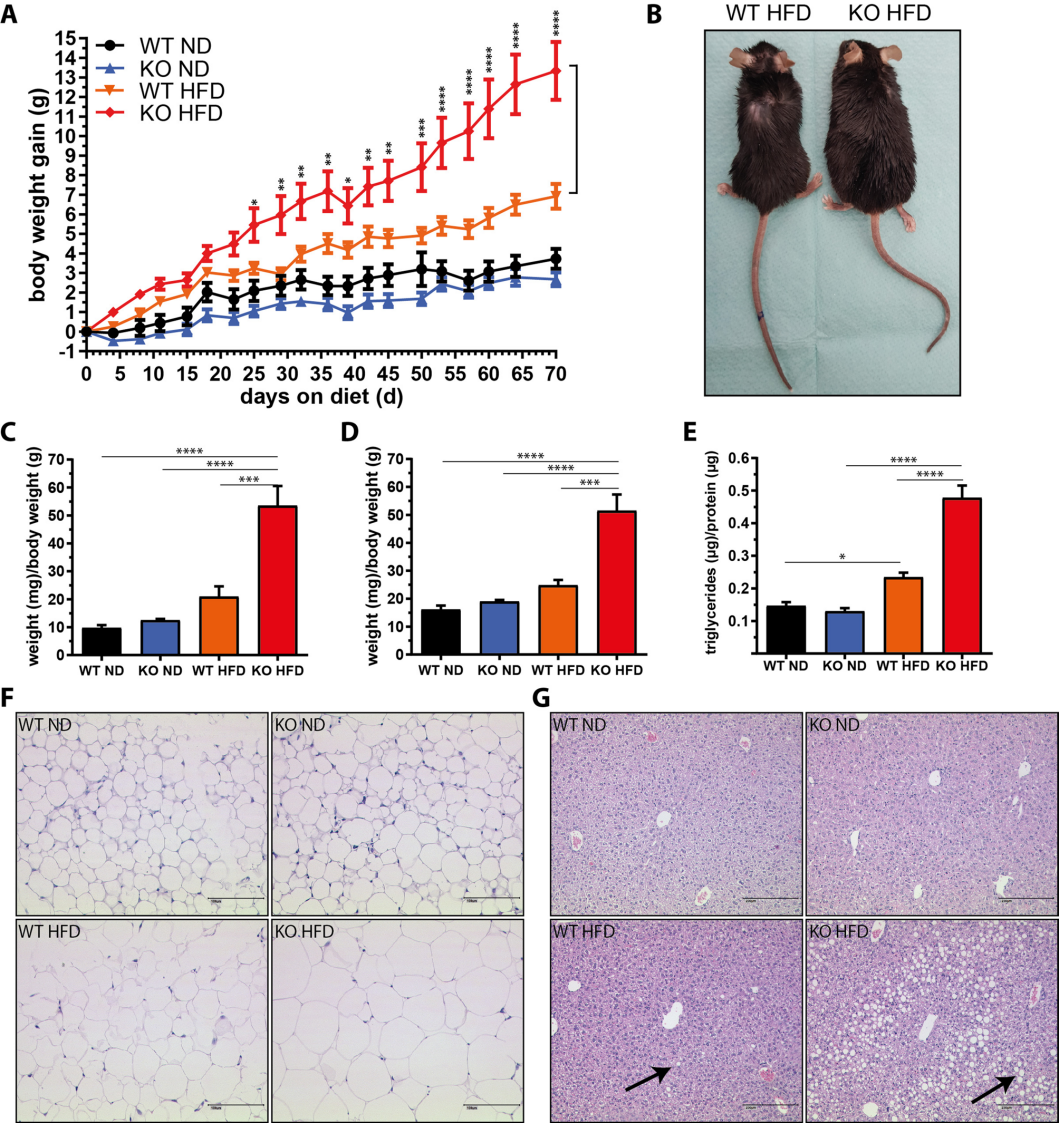
Increased body weight, fat pad mass, and liver triglyceride accumulation in miR-146a^−/−^ mice on a high-fat diet. Female miR-146a^−/−^ (KO) and respective control mice (WT) at an age of 10 weeks were fed a high-fat (HFD) or respective normal diet (ND) for 10 weeks. **a** Body weight gain. **b** Representative photographs of WT and KO mice on HFD. **c** Gonadal WAT (gWAT) weight and (**d**) inguinal WAT (iWAT) weight relative to body weight. **e** Liver triglyceride content in relation to total protein. **f** and **g** Representative microphotographs of H&E stained gWAT and liver sections. Arrows indicate liver fat vacuoles. Data are displayed as mean and SEM of 10 (**a**) or 5 (**c**–**e**) animals per group. Statistics: (**a**) two-way ANOVA with Bonferroni correction, (**c**–**e**) one-way ANOVA with Tukey correction. *p < 0.05, **p < 0.01, ***p < 0.001, ****p < 0.0001

MiR-146a^−/−^ and control mice were fed a high-fat diet (HFD) or a respective control diet (normal diet, ND). As expected, control animals gained more weight upon HFD compared to ND feeding (Fig. 1a and Supplemental Fig. S2). While the weight of miR-146a^−/−^ mice was comparable to that of controls on a ND, they gained significantly more weight on an HFD (Fig. 1a, b and Supplemental Fig. S2). In line, the weight of gonadal and inguinal white adipose tissues (gWAT and iWAT) was significantly increased in miR-146a^−/−^ mice (Fig. 1c, d) along with an increase in adipocyte size (Fig. 1f and Supplemental Fig. S3). Furthermore, miR-146a^−/−^ mice displayed clear signs of liver steatosis after 10 weeks of HFD. Histological analysis revealed intermediate steatosis of the liver parenchyma (Fig. 1g) and the levels of liver triglycerides were significantly increased (Fig. 1e). There was no evidence of inflammatory cell infiltration into either adipose tissues or liver (Fig. 1f, g and Supplemental Fig. S3).

### High-fat diet induces marked insulin resistance in miR-146a^−/−^ mice

**Fig. 2:**
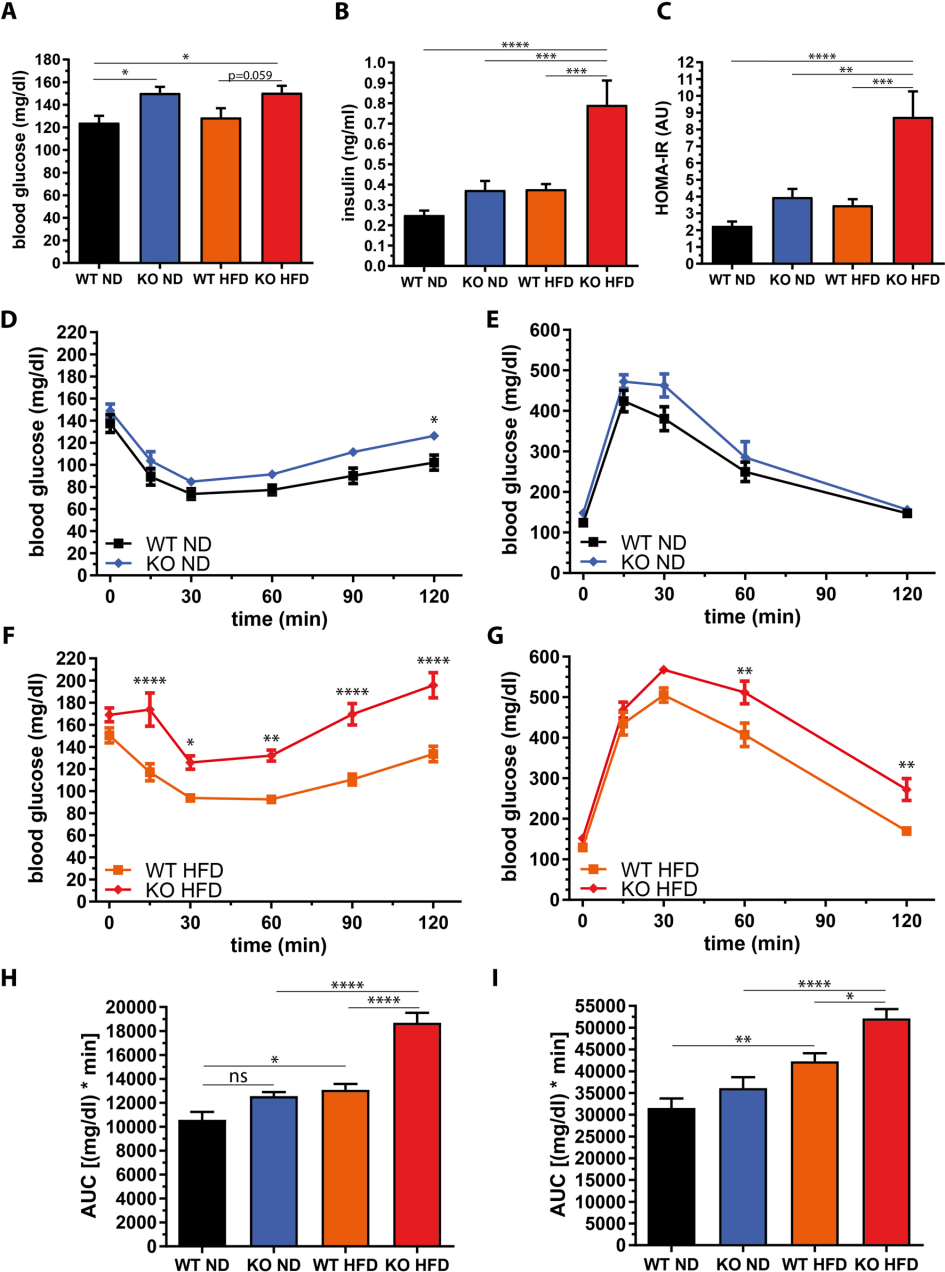
Decreased insulin sensitivity and glucose tolerance in miR-146a^−/−^ mice on a high-fat diet. Blood samples of female miR-146a^−/−^ (KO) and respective control mice (WT) were analyzed after 10 weeks of high-fat diet (HFD) or normal diet (ND) and the animals’ insulin and glucose metabolism were assessed. **a** Fasted blood glucose, **b** fasted plasma insulin, and **c** HOMA-IR. **d** and **e** Insulin tolerance test (ITT) and oral glucose tolerance test (OGTT) in mice fed a ND. **f** and **g** ITT and OGTT in mice fed an HFD. **g** Area under the curve (AUC) of ITT. **h** AUC of OGTT. Data are displayed as mean and SEM of 10 animals per group. Statistics: (**a**, **b**, **c**, **h**, **i**) one-way ANOVA with Tukey correction, (**d**–**g**) two-way ANOVA with Bonferroni correction, ns not significant, *p < 0.05, **p < 0.01, ***p < 0.001, ****p < 0.0001

Glucose and insulin as well as the resulting HOMA-IR index, a measure of insulin resistance, were significantly increased in miR-146a^−/−^ mice on an HFD (Fig. 2a, b, c). On an ND, miR-146a^−/−^ mice showed slightly impaired insulin sensitivity and glucose tolerance (Fig. 2d, e, h, i). On an HFD, however, they exhibited severe insulin resistance and glucose intolerance (Fig. 2f–i). Data obtained after 5 weeks of diet underlined the aggravation of the metabolic phenotype over time and are given in Supplemental Fig. S4.

### Myeloproliferation in miR-146a^−/−^ mice is not affected by HFD

The initial description of the mouse model reported gross anatomy to be normal in young mice [9]. However, starting at an age of 6–8 months, mice developed a spontaneous autoimmune disorder with splenomegaly, lymphadenopathy, and multiorgan inflammation, eventually resulting in premature death [9]. We performed our experiments at a younger age and analyzed mice for differences in the leukocyte compartment, and controlled for the inflammatory phenotype as well as the impact of HFD feeding.

**Fig. 3:**
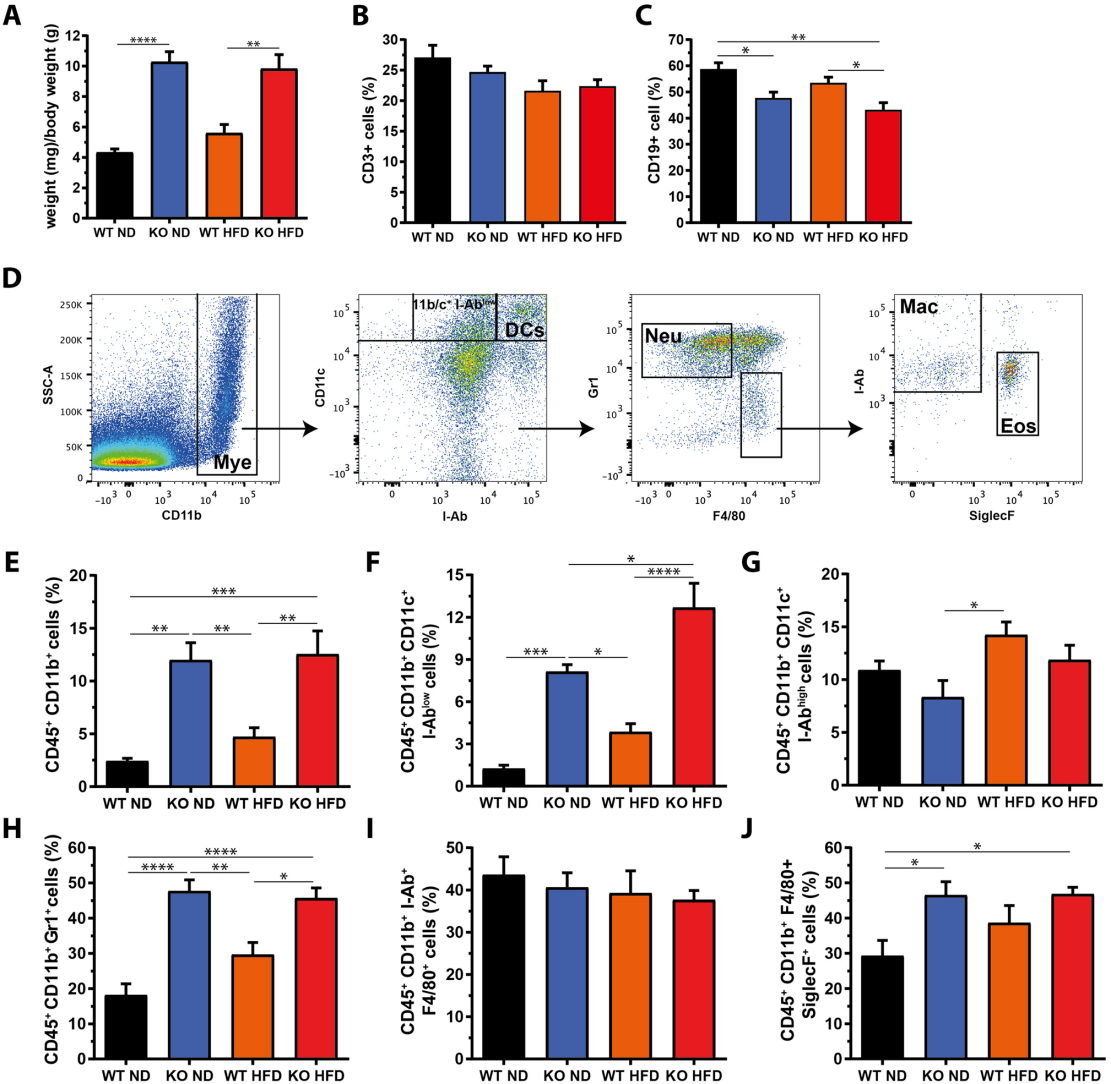
Myeloproliferation in miR-146a^−/−^ mice is not affected by high-fat diet. After 10 weeks of high-fat diet (HFD) or respective normal diet (ND), miR-146a^−/−^ (KO) or respective control mice (WT) were sacrificed and splenocytes were isolated and analysed by flow cytometry. **a** Spleen weight relative to body weight. **b** T cells (CD3^+^) and **c** B cells (CD19^+^). **d** Gating strategy for myeloid-derived subpopulations. **e** Myeloid cells (Mye), **f** CD11b^+^/c^+^ I-Ab^low^ cells, **g** CD11b^+^/c^+^ dendritic cells (DCs), **h** neutrophils (Neu), **i** macrophages (Mac), and **j** eosinophils (Eos). Data are displayed as mean and SEM of 5 animals per group. Statistics: (**a**–**c**, **e**–**j**) one-way ANOVA with Tukey correction. *p < 0.05, **p < 0.01, ***p < 0.001

At an age of 20 weeks, miR-146a^−/−^ mice had an increased spleen weight, which was not influenced by the diet regime (Fig. 3a). Splenocytes were isolated and subjected to flow cytometry to quantify the major immune cell populations. The percentage of CD3^+^ T cells was comparable between genotypes and diets (Fig. 3b). The percentage of CD19^+^ B cells was reduced by ~ 19% in miR-146a^−/−^ mice compared to controls, but this was independent of the diet (Fig. 3c). Moreover, in line with the original description, miR-146a^−/−^ mice developed myeloproliferation as measured by an increased percentage of CD11b^+^ cells, which was also not influenced by diet (Fig. 3d, e). We further characterized the myeloid-derived subpopulations and found that dendritic cells (CD11b^+^/c^+^, I-Ab^high^, Fig. 3g) were not significantly altered, but a CD11b^+^/c^+^, I-Ab^low^ subpopulation, which might represent dendritic precursor cells (Fig. 3f) was increased in the miR-146a^−/−^ animals. A comparable pattern was seen for the Gr1^+^ neutrophil population (Fig. 3h). The quantity of F4/80^+^ macrophages (Fig. 3i) was not altered in the spleens and eosinophils (SiglecF^+^, Fig. 3j) were slightly increased in the miR-146a^−/−^ animals. From this set of data, we conclude that miR-146a deficiency causes splenomegaly due to myeloproliferation already at an age of 5 months, without this phenotype being aggravated by HFD feeding.

### miR-146a^−/−^ mice show no increased immune cell infiltration into WAT

**Fig. 4:**
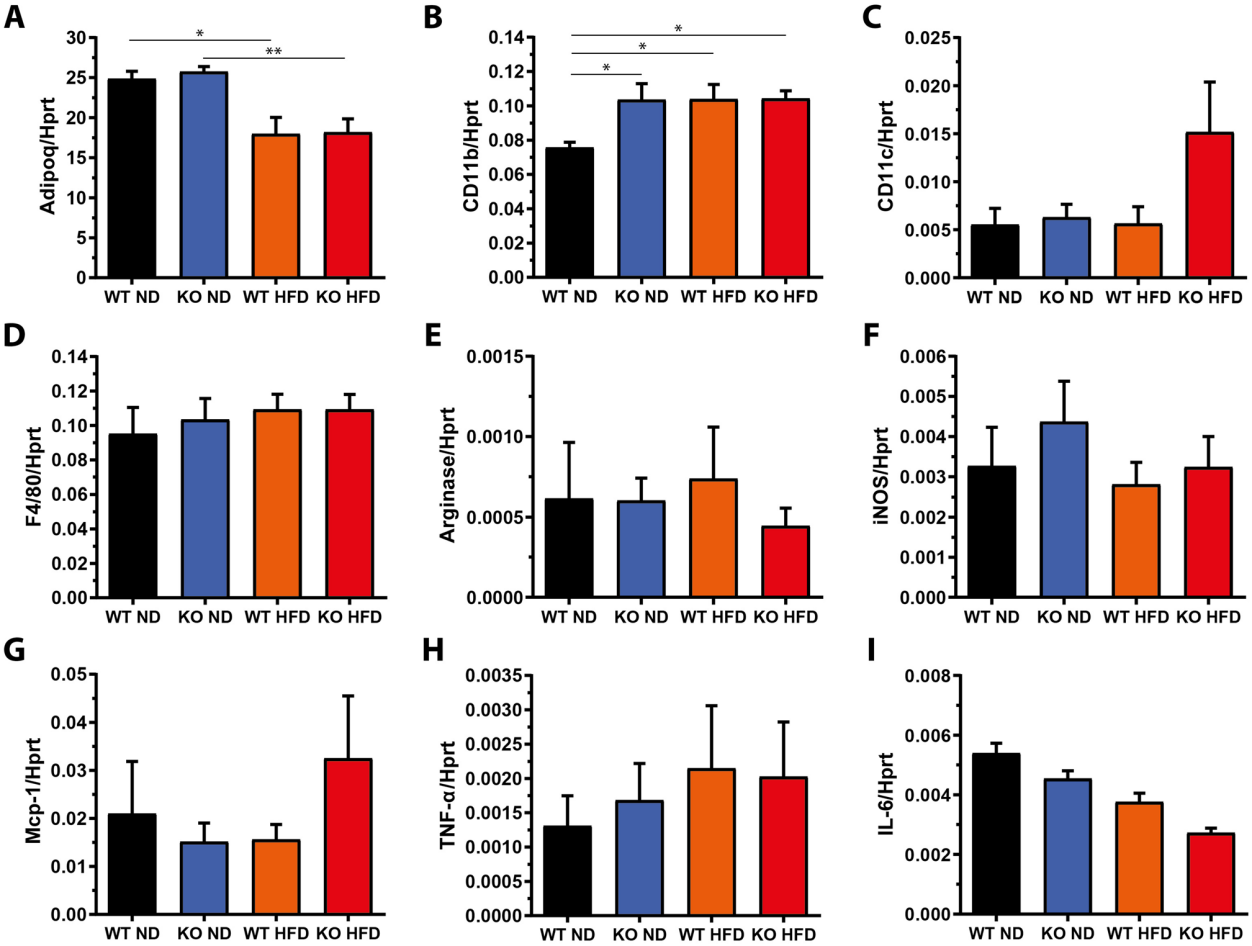
Markers for infiltrating immune cells and WAT inflammation are not increased in miR-146a^−/−^ mice on a high-fat diet. After 10 weeks of high-fat (HFD) or normal diet (ND) feeding miR-146a^−/−^ (KO) or respective control mice (WT) were sacrificed, gonadal fat pads (gWAT) were dissected and processed for qPCR. mRNA expression is given in relation to Hprt as reference gene (2^−ΔCT^). **a** Adiponectin (Adipoq), **b** cluster of differentiation 11b (Cd11b), **c** cluster of differentiation 11c (Cd11c), **d** EGF-like module-containing mucin-like hormone receptor-like 1 (F4/80), **e** arginase, **f** iNOS, **g** monocyte chemoattractant protein 1 (Mcp-1), **h** tumor necrosis factor α (Tnf-a), and **i** interleukin 6 (IL-6) expression. Data are displayed as mean and SEM of 5 animals per group. Statistics: One-way ANOVA with Tukey correction. *p < 0.05, **p < 0.01

Runtsch et al*.* reported a significant increase of immune cell markers, especially those of macrophages, and pro-inflammatory factors in WAT of miR-146a^−/−^ mice upon HFD feeding [8]. We, thus, measured a panel of inflammation markers in our experiment. We detected a downregulation of adiponectin, which exerts anti-inflammatory functions and represents an indicator of insulin sensitivity, and an upregulation of the myeloid marker CD11b, but not CD11c or F4/80, in gWAT of control mice on an HFD (Fig. 4). There were no statistically significant differences in the mRNA expression the studied markers (CD11b, CD11c, F4/80, Arg-1, iNOS, Tnf-a, Il-6, Mcp-1) between control and miR-146a^−/−^ mice on an HFD (Fig. 4). This agrees well with histological analyses which revealed an absence of inflammatory infiltrates (Fig. 1c). Comparable results were obtained for iWAT (Supplemental Fig. S5), except for CD11c which was higher in miR-146a^−/−^ compared to control mice upon HFD feeding. Importantly, HFD itself did not further increase the presence of CD11c^+^ cells in iWAT of miR-146a^−/−^ mice. The lack of striking differences in adipose inflammation between miR-146a^−/−^ and control mice and between miR-146a^−/−^ mice fed either ND or HFD suggests that local inflammatory processes within WAT did not contribute to the systemic metabolic deterioration observed in obese miR-146a^−/−^ animals.

The bona fide targets of miR-146a, Irak1 and Traf6, are responsible for the age-dependent immunoproliferative phenotype of miR-146a^−/−^ mice [9]. It was also proposed that an upregulation of Traf6 in macrophages might be responsible for a decreased insulin sensitivity of miR-146a^−/−^ mice [8]. Liao et al*.* reported a cell-intrinsic effect of miR-146a in murine 3T3-L1 adipocytes by the downregulation of Traf6 [11]. However, in our experiments, neither Traf6 nor Irak1 showed a relevant difference in mRNA and protein expression in iWAT or gWAT between miR-146a^−/−^ and control mice upon ND feeding (Supplemental Figs. S6 and S7). In gWAT of animals on an HFD, the mRNA expression of both genes was lower in miR-146a^−/−^ mice compared to controls although an upregulation would be expected if they were relevant targets of miR-146a in this context (Supplemental Fig. S6). Protein expression of both genes was unchanged under all conditions, except for a higher expression of Irak1 upon HFD in iWAT in both genotypes (Supplemental Fig. S7). Thus, the expression level of Traf6 and Irak1 in WAT appears to be mostly irrelevant to the observed metabolic phenotype of miR-146a^−/−^ mice. To corroborate this assumption in vitro, we measured the expression of both genes in SGBS cells overexpressing miR-146a and found IRAK1 significantly downregulated, while TRAF6 was not altered (Supplemental Fig. S8A). To finally rule out a potential role in cellular insulin sensitivity, we knocked down IRAK1 expression using siRNA. We achieved a knockdown by 67%, but neither basal nor insulin-stimulated glucose uptake was affected by the reduced level of IRAK1 (Supplemental Fig. S8 B, C). Taking all these findings together, we conclude that TRAF6 and IRAK1 are not major mediators of the miR-146a effect on insulin sensitivity.

### Increased insulin sensitivity in adipocytes overexpressing miR-146a mimic

**Fig. 5:**
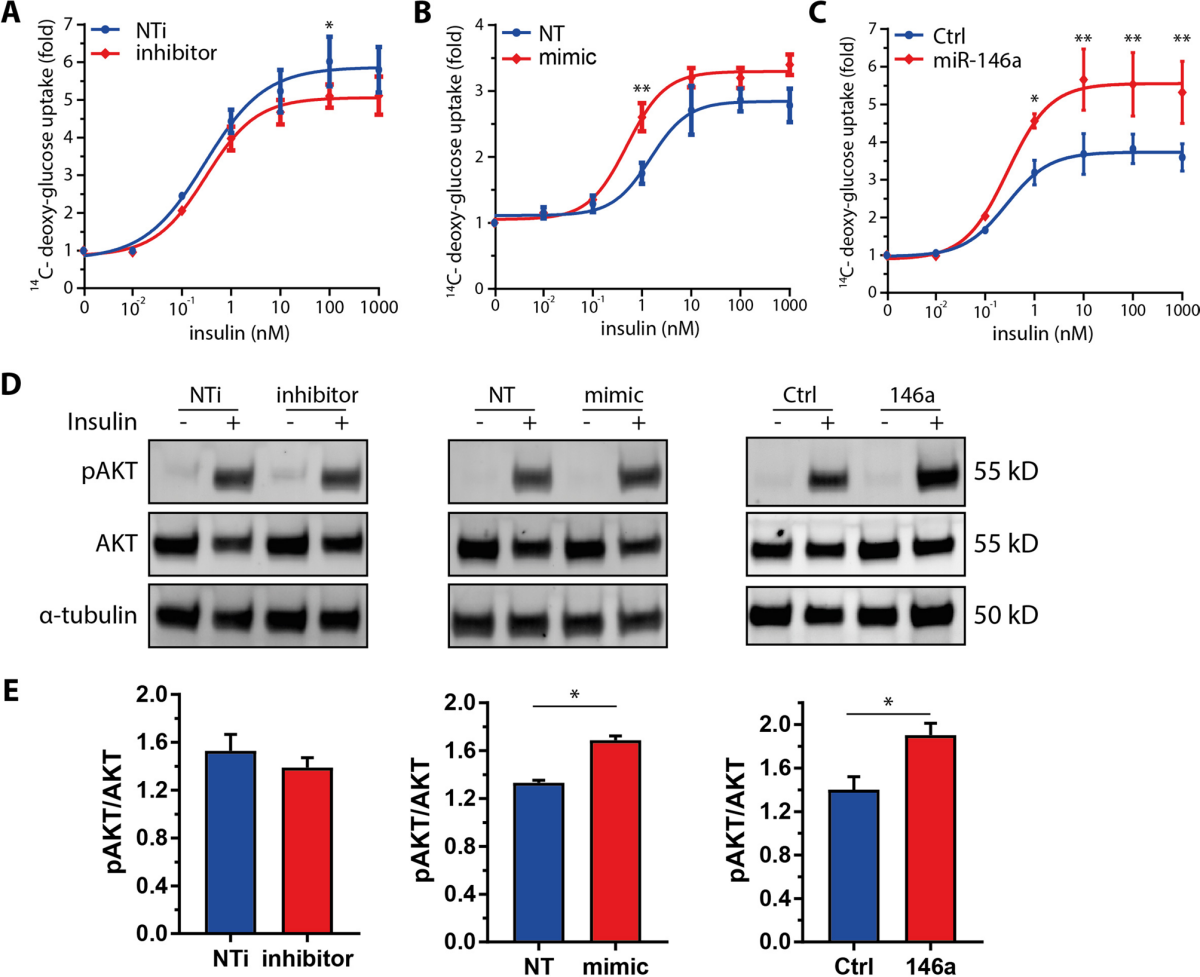
miR-146a regulates insulin-stimulated glucose uptake in adipocytes. To assess the influence of miR-146a on insulin sensitivity in vitro*,* glucose uptake experiments were performed with SGBS adipocytes. The amount of glucose taken up by the cells was measured by scintillation counting and normalized to the basal glucose uptake (0 nM insulin) and phosphorylation of AKT was assessed. **a** miR-146a inhibitor (50 nm) or inhibitor control (NTi, 50 nM) transfected adipocytes. **b** miR-146a mimic (20 nM) or control (NT, 20 nM) transfected adipocytes. **c** Adipocytes stably overexpressing miR-146a or non-target control (Ctrl). **d** Representative Western blot for pAKT (Ser473) and AKT after 15 min stimulation with 0 nM and 10 nM insulin with α-tubulin as loading control. **e** Densitometric analysis of 4 independent experiments displayed as mean and SEM with phosphorylated AKT (Ser473) normalized to total AKT. Data are displayed as mean and SEM of 3 (**a** and **c**) or 4 (**b**) independent experiments. Statistics: (**a**, **b**, **c**) two-way ANOVA with Bonferroni correction and non-linear fit with four parameters, (**e**) paired *t* test, *p < 0.05, **p < 0.01

We consequently hypothesized that miR-146a has an inflammation-independent regulatory effect on the insulin sensitivity of adipocytes. To study possible cell-autonomous effects of miR-146a on adipocyte function we used the human SGBS cell strain as a model system. SGBS cells were originally derived from subcutaneous WAT of a patient with Simpson-Golabi-Behmel syndrome [12]. They have a high capacity for adipogenic differentiation ex vivo and display the cellular characteristics of human primary adipocytes [12, 13]. SGBS cells were transfected with a miR-146a inhibitor or control oligonucleotide (NTi) and their insulin sensitivity was investigated in a glucose uptake assay (Fig. 5a). Consistent with our hypothesis and the in vivo data, adipocytes transfected with the miR-146a inhibitor showed a lower response to insulin compared to control cells (Fig. 5a). Vice versa, adipocytes transfected with a miR-146a mimic displayed significantly increased insulin sensitivity compared to cells transfected with a control oligonucleotide (NT) (Fig. 5b). To corroborate these findings, we also generated SGBS cells with a stable overexpression of miR-146a. To this end, we cloned the miR-146a sequence and its flanking genomic region into a lentiviral expression vector. MiR-146a expression levels are given in Supplemental Fig. S12. SGBS adipocytes stably transduced with the resulting miR-146a lentivirus displayed an almost twofold increase in glucose uptake (Fig. 5c). This effect compares easily to the insulin-sensitizing effect of FGF-21 [14] and should thus be considered clinically relevant. Additionally, Ser473 phosphorylation of AKT was significantly increased upon insulin stimulation with both gain-of-function approaches (Fig. 5d, e).

### NPR3 is a target of miR-146a

**Fig. 6:**
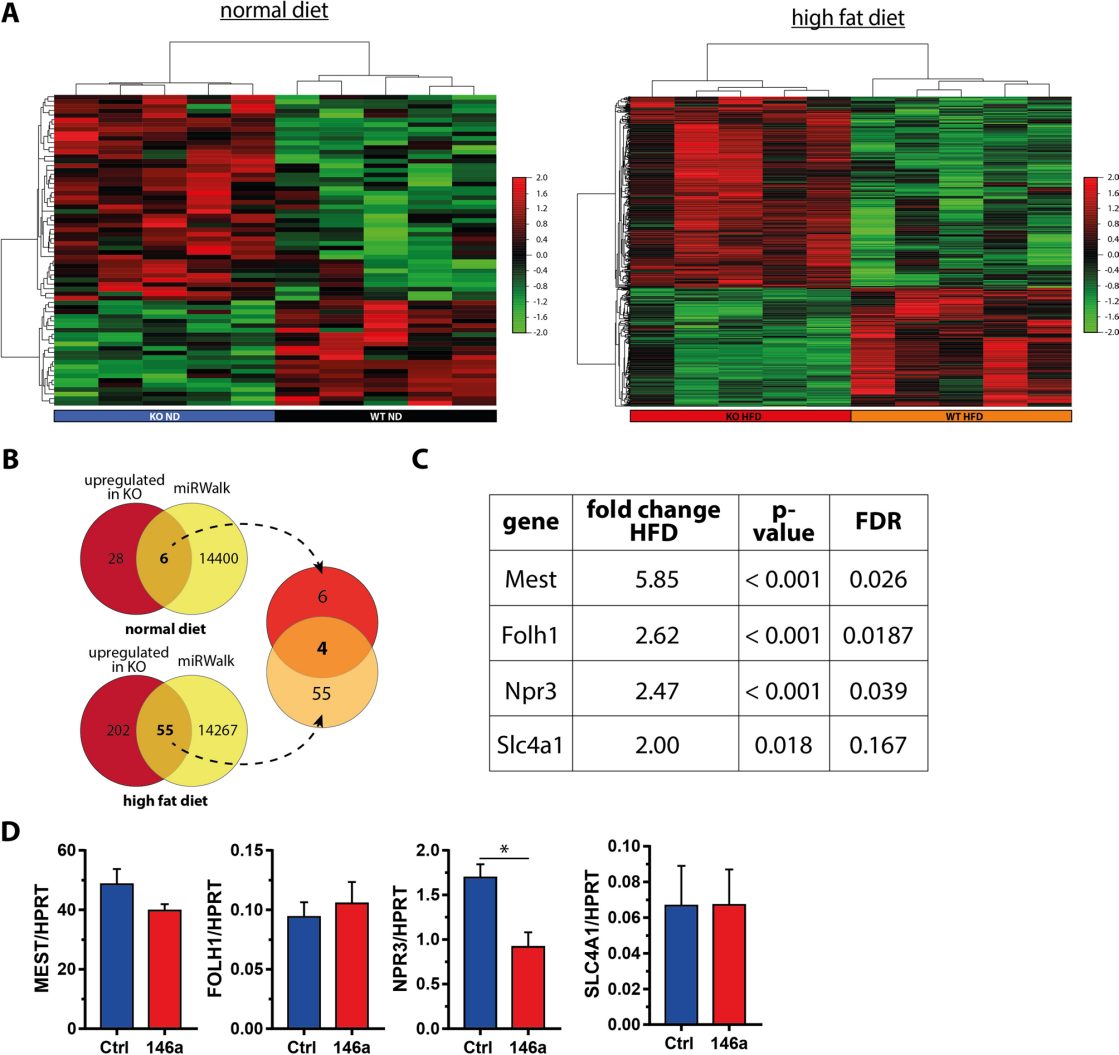
miR-146a target identification. **a** Hierarchical cluster analysis of genes differentially expressed between miR-146a^−/−^ (KO) and control (WT) samples with 5 mice per group (average linkage clustering, 1-correlation). **b** Schematic illustration of miR-146a target identification with Affymetrix microArray-based transcriptome analysis and miRNA target prediction. RNA was isolated from gonadal WAT and processed for gene expression analysis. The upregulated genes in miR-146a^−/−^ samples (red) in both diet groups were compared to the predicted targets of miR-146a in the database miRWalk (yellow). Regulated and predicted genes from both diets (normal diet: 6, high-fat diet: 55) were compared and 4 candidate genes, which are upregulated in miR-146a^−/−^ mice and predicted as miR-146a targets, were identified. These genes are given in (**c**) with the fold change to wild-type mice. **d** Validation of the candidate genes in SGBS adipocytes overexpressing miR-146a. Data are displayed as mean and SEM of 4 independent experiments. Statistics: (**d**) paired *t* test, *p < 0.05

**Fig. 7:**
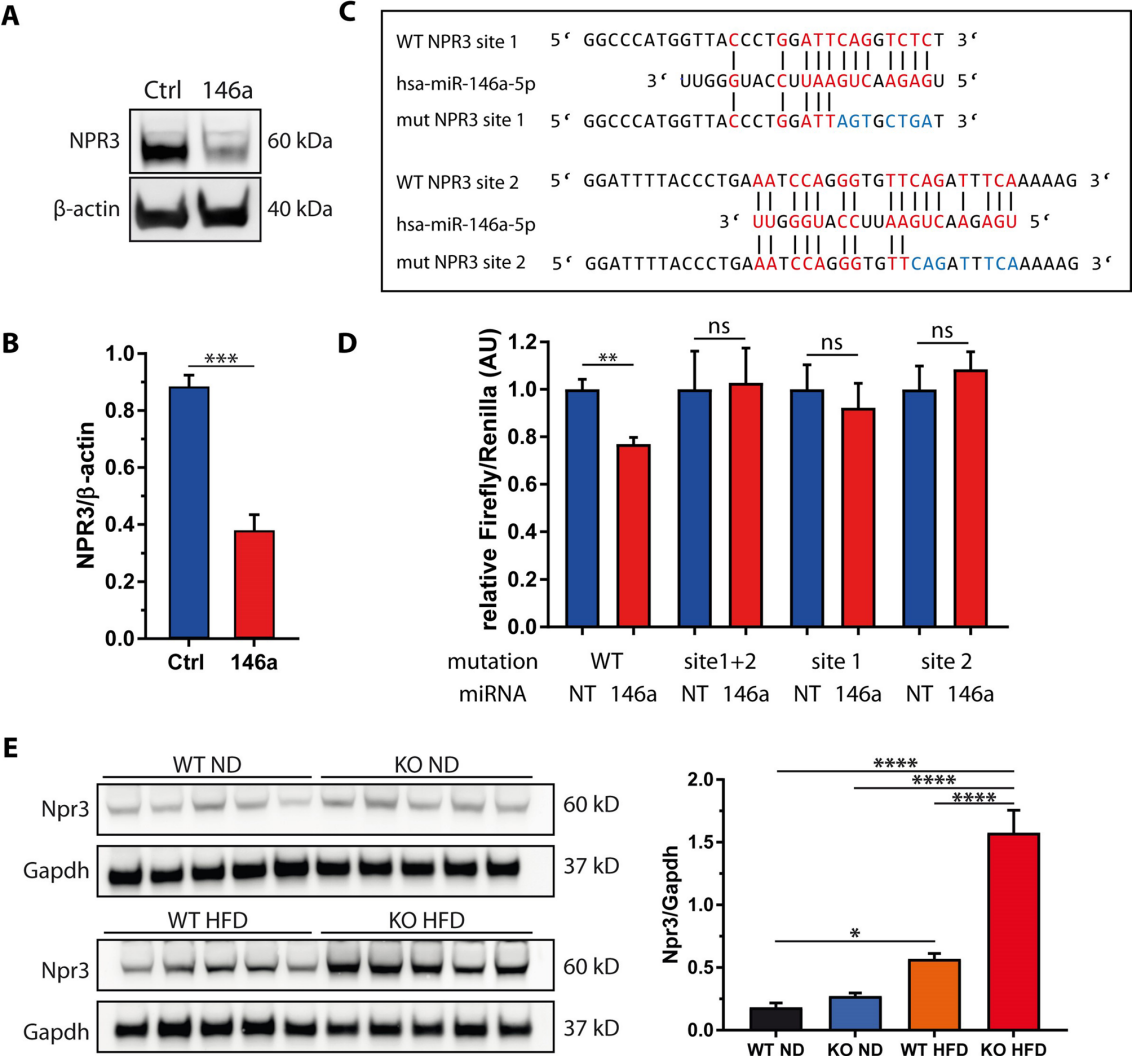
NPR3 is a target gene of miR-146a in human adipocytes and murine WAT. **a** Representative Western blot for NPR3 in SGBS adipocytes overexpressing miR-146a (146a) compared to control cells (Ctrl) with (**b**) densitometric analysis of 4 independent experiments in relation to β-actin. **c** Illustration of miR-146a binding sites in the NPR3 3′ UTR predicted by miRWalk 3.0. *WT* wild type, *mut* mutated. **d** Firefly luciferase signal normalized to Renilla luciferase signal of HEK293 cells co-transfected with miR-146a mimic or control (NT) and pmirGLO dual-luciferase plasmid carrying either the two predicted NPR3-miR-146a binding sites (WT), a plasmid with both target sites (site 1 + 2), or a plasmid with either binding site 1 (site 1) or binding site 2 (site 2) mutated. **e** Npr3 protein expression in gonadal fat pads of miR-146a^−/−^ (KO) and control (WT) mice after 10 weeks of high-fat diet (HFD) or normal diet (ND) with densitometric analysis of 5 animals per group displayed as mean and SEM. Statistics: (**b** and **d**) paired *t* test, **e** one-way ANOVA with Tukey correction *p < 0.05, **p < 0.01, ***p < 0.001, ****p < 0.0001

We next aimed to identify target genes mediating the effect of miR-146a on insulin sensitivity in adipocytes. To this end, we performed an Affymetrix microArray-based transcriptome analysis comparing gWAT of miR-146a^−/−^ and control mice under both diet regimes. 68 genes were differentially expressed on ND and 516 on HFD, respectively (fold change > 1.5, *p* < 0.05) (Fig. 6a and Supplemental Table 1). Out of these, 34 (ND) and 257 (HFD) genes were upregulated in miR-146a^−/−^ mice and therefore represented potential miR-146a target genes (Fig. 6a). We then performed an in silico target prediction analysis using miRWalk 3.0 [15]. The search term *hsa-miR-146a-5p* retrieved 14,406 hits for genes with potential miR-146a target sites. An intersection analysis revealed that 6 (ND) and 55 (HFD) genes from the array analysis were predicted as direct miR-146a targets and significantly upregulated in the KO animals as displayed in Venn diagrams (Fig. 6b). Four genes were upregulated under both dietary regimes. Mest, Folh1, Npr3, and Slc4a1 showed an upregulation of ≥ 2.0-fold in the HFD group (Fig. 6c). As we assumed a cell-autonomous effect in adipocytes, we decided to assess the expression of these candidates in SGBS adipocytes stably overexpressing miR-146a. While MEST, FOLH1, and SLC4A1 were not differentially expressed, NPR3 was significantly downregulated on the mRNA level (Fig. 6d). This finding was confirmed on the protein level as expression of NPR3 was reduced by ~ 58% upon miR-146a overexpression (Fig. 7a, b). Vice versa and validating the array data, the RNA and protein expression of Npr3 was significantly upregulated in both gWAT and iWAT of miR-146a^−/−^ mice compared to controls (Fig. 7e and Supplemental Fig. S9). Furthermore, a reporter gene assay harboring the predicted miR-146a target sites in the 3’ UTR of the gene, verified NPR3 as a direct target of miR-146a (Fig. 7c, d). Both miRNA-binding sites predicted by miRWalk 3.0 seem to be equally relevant as the insertion of mutations in the binding sites 1 and 2 or both reversed the miR-146a-induced down-regulation of the Firefly/Renilla signal. Taken together, our set of in silico, in vitro, and in vivo data identified NPR3 as a target gene of miR-146a in adipocytes and WAT as a whole.

### NPR3 regulates insulin-induced glucose uptake and de novo lipogenesis

**Fig. 8:**
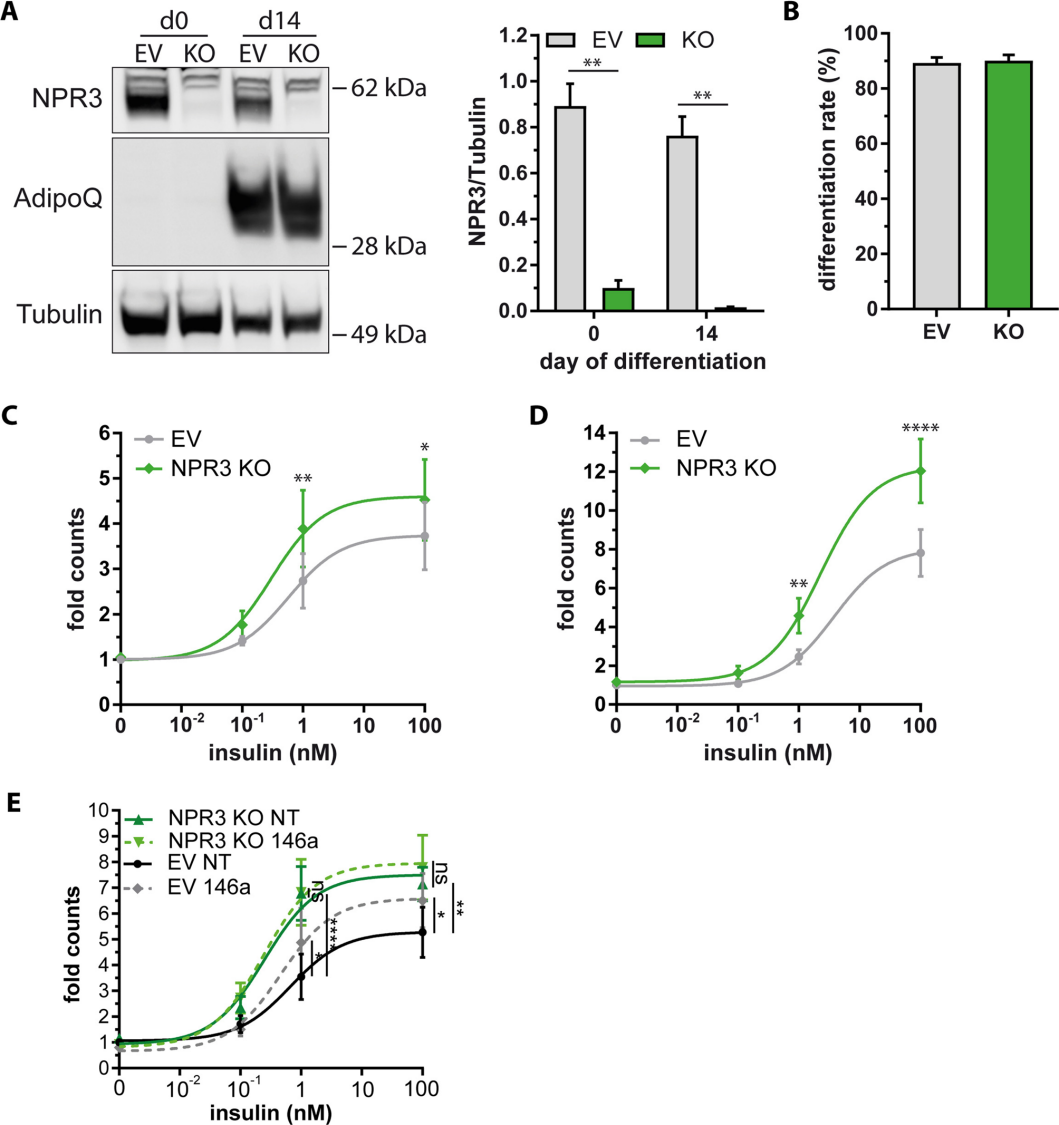
CRISPR/Cas9-mediated NPR3 ablation increases insulin-stimulated glucose uptake and de novo lipogenesis. **a** Representative Western blot for NPR3 (60 kD) and adiponectin (AdipoQ) in SGBS pre-adipocytes (d0) and adipocytes (d14) in control cells (EV) and NPR3 KO cells with densitometric analysis displayed as mean and SEM of 4 independent experiments in relation to tubulin. **b** Differentiation rates of adipocyte cultures (adipocytes/total cells). **c** Insulin-dependent glucose uptake was measured by scintillation counting and normalized to the basal rate of control cells (0 nM insulin). **d** Insulin-dependent de novo lipogenesis was measured by scintillation counting and normalized to the basal rate of control cells (0 nM insulin). **e** Insulin-dependent glucose uptake in control (EV) and NPR3 KO cells transfected with non-targeting (NT) or miR-146a mimic was measured by scintillation counting and normalized to the basal rate of EV cells (0 nM insulin). Data are displayed as mean and SEM of 5 (**c**) and 4 (**d**) independent experiments. Statistics: (**a**) two-way ANOVA with Bonferroni correction (**c** and **d**) two-way ANOVA with Bonferroni correction and non-linear fit with three parameters. *p < 0.05, **p < 0.01, ****p < 0.0001

We then came forward with the hypothesis that NPR3 regulates insulin sensitivity in adipocytes and decided to generate NPR3-deficient adipocytes using CRISPR/Cas9. We achieved a downregulation of NPR3 protein levels by ~ 87% in bulk cultures of SGBS preadipocytes and ~ 92% in adipocytes, respectively (Fig. 8a). Importantly, adipogenesis was not affected by the absence of NPR3 as indicated by comparable levels of adiponectin mRNA and protein as well as equal differentiation rates (Fig. 8a, b and Supplemental Fig. S10). Most strikingly and confirming our hypothesis, the knockout of NPR3 resulted in a robust increase in insulin-stimulated glucose uptake (Fig. 8c) as well as insulin-stimulated de novo lipogenesis (Fig. 8d). Finally, to prove that that NPR3 is the main miR-146a target mediating the insulin-sensitization we transfected NPR3-deficient cells with either with non-targeting control (NT) or miR-146a mimic and performed glucose uptake experiments. Also in this set of experiments, miR-146a enhanced the insulin-stimulated uptake of glucose into empty vector carrying adipocytes. Importantly, there was no further improvement upon miR-146a transfection if NPR3-deficient cells (Fig. 8e), suggesting that NPR3 is indeed the main target gene mediating the effects of miR-146a on insulin sensitivity.

## Discussion

miRNAs are regarded as a breakthrough technology in pharmaceutical development [16]. Also in the field of metabolism, obesity, and related disorders, miRNAs have gained considerable attention [17, 18]. miR-375 for instance was identified as crucial regulator of insulin secretion in pancreatic islets [19] and miR-103 and miR-107 were among the first miRNAs demonstrated to play important roles in the regulation of insulin sensitivity [20]. MiR-146a has long been suspected to contribute to the development and consequences of type 1 and 2 diabetes. It was for example reported that miR-146a is involved in the pathogenesis of diabetic complications. In a model of streptozotocin (STZ)-induced diabetes, miR-146a^−/−^ mice proved more prone to develop diabetic nephropathy with exacerbated proteinuria, renal macrophage infiltration, and glomerular hypertrophy [21, 22]. Vice versa*,* under similar circumstances transgenic overexpression of miR-146a in endothelial cells prevented inflammation-induced damage to the heart, retina, and kidneys [23, 24]. Along the same line, the systemic delivery of miR-146a mimics reduced peripheral diabetic neuropathy in B6.*db/db* mice, a common model of type 2 diabetes [25]. Furthermore, systemic miR-146a delivery decreased body weight gain in a model of diet-induced obesity [26]. Runtsch et al*.* showed that miR-146a is involved in the determination of systemic insulin sensitivity and glucose homeostasis [8]. Clinical data further support such effects miR-146a on insulin sensitivity and glucose tolerance. A single-nucleotide polymorphism in the miR-146a gene, the rs2910164 variant, is more abundant among type 2 diabetic compared to non-diabetic subjects, suggesting a causal role in pathogenesis of the disease. In silico analyses predicted that the according base exchange of guanine to cytosine causes structural instability of pre-miR-146a [27]. This might lead to a reduction of mature miR-146a and, therefore, to a decrease in function. Balasubramanyam et al*.* measured the expression levels of miR-146a in peripheral blood monocytes from patients with type 2 diabetes and non-diabetic controls and found miR-146a levels to be significantly reduced in diabetics [28]. This study and others were recently summarized in a meta-analysis demonstrating a tight association of reduced miR-146a expression with type 2 diabetes susceptibility [29].

In our hands, miR-146a^−/−^ mice challenged with an HFD showed an aggravated weight gain, higher fat mass, and exacerbated insulin resistance and glucose intolerance accompanied by pronounced liver steatosis, confirming published data [8]. Interestingly, we could not detect signs for enhanced inflammatory processes in WAT in miR-146a^−/−^ animals fed an HFD. This is in line with observations of Javidan et al*.* [30], but in contrast to those of Runtsch et al*.* describing an increased accumulation of macrophages in adipose tissue and an altered gene expression pattern in macrophages as the underlying cause for the metabolic phenotype of miR-146a^−/−^ mice [8]. Adipose tissue inflammation is regarded a key event in the development of obesity-induced insulin resistance, but our histological studies and transcriptome analysis revealed no signs of increased WAT inflammation in miR-146a^−/−^ mice, neither on ND nor HFD. We, therefore, suggest that other, inflammation-independent processes are involved in the development of insulin resistance and glucose intolerance upon miR-146a deficiency and propose a direct action of miR-146a on metabolic pathways in adipocytes. Concordantly, inhibiting the function of miR-146a in vitro in SGBS adipocytes resulted in reduced insulin-stimulated glucose uptake, whereas augmenting its function led to an increase.

Exploiting transcriptome analysis of gWAT, we identified NPR3 as differentially expressed between miR-146a^−/−^ and control mice, both under ND and HFD. Indeed, our study demonstrated for the first time that NPR3 is a direct target gene of miR-146a, both in vivo and in vitro. Most importantly, CRISPR/Cas9-mediated knockout of NPR3 in adipocytes resulted in a significant increase in insulin-stimulated glucose uptake and de novo lipogenesis, identifying NPR3 as important regulator of insulin sensitivity.

NPR3 is one out of three known receptors for natriuretic peptides [31]. Natriuretic peptides (NPs) are the main antagonists of the renin–angiotensin–aldosterone system (RAAS). Their family includes the atrial natriuretic peptide (ANP) and the natriuretic peptides of the B-type (BNP) and C-type (CNP). Natriuretic peptide receptor 1 (NPR1) and natriuretic peptide receptor 2 (NPR2) are transmembrane guanylyl cyclases. Both receptors catalyze the synthesis of cGMP, which mediates the best known effects of NPs [31]. The natriuretic peptide receptor 3 (NPR3) removes NPs from the circulation by receptor-mediated internalization and degradation. However, signal functions for NPR3 are also predicted, including inhibition of adenylyl cyclase and activation of phospholipase C [31]. NPs and their receptors regulate natriuresis and diuresis and smooth muscle relaxation [32]. Several studies suggest that NPs may also influence the systemic and adipose tissue metabolism via auto-, para-, and endocrine mechanisms. Patients with obesity, insulin resistance, and type 2 diabetes show reduced plasma levels of ANP and BNP [32]. Low ANP levels represent an established biomarker for the progression of type 2 diabetes [33]. NPs also play a role in the regulation of body weight and energy metabolism by increasing the capacity of fat oxidation and mitochondrial biogenesis and, thus, reducing the harmful effects of a high-fat diet [34–36]. An increased expression of NPR3 in WAT of obese patients was associated with lower levels of bioavailable NPs and consequently to a progression of obesity and typical concomitant diseases, such as diabetes, hypertension, and liver steatosis [32, 37]. The specific role of NPR3 in WAT was just recently demonstrated [38]. The knockout of NPR3 in adipocytes, but not in skeletal muscle, resulted in a resistance to diet-induced obesity, increased energy expenditure, improved insulin sensitivity, increased glucose uptake, as well as protection from liver steatosis and visceral fat inflammation [38]. This is in line with our data showing an increase in body weight on HFD, a decreased insulin sensitivity and glucose tolerance upon NPR3 upregulation due to miR-146a deficiency. Furthermore, ANP and BNP can increase glucose uptake both in the presence and absence of insulin [39]. This leads us to two explanations how the knockout of NPR3 increases insulin sensitivity in adipocytes. First, NPR3 might act as a clearance receptor. Its absence would increase the amount of bioavailable NP, which in turn influence insulin sensitivity via NPR1 and NPR2. Second, NPR3 itself might exert signaling functions. Its absence might cause intracellular signaling changes and lead to insulin sensitization. Further investigations are required to corroborate these suggestions.

It is intriguing that, although mice from the same supplier with the same genetic background were used, the studies investigating the impact of miR-146a on obesity showed some overlap but on the other hand also distinct findings [8, 30]. We speculate that additional, extrinsic factors may contribute to the development of the specific phenotypes and propose microbiota differences as possible explanation. It is well established that the microbiome is crucial for the reproducibility of in vivo data in all research fields, including those of obesity and diabetes [40, 41]. Animals from the same source can acquire different microbiotic colonization in different animal facilities which in turn can result in distinct phenotypes [40–42]. Most importantly though, miR-146a deficiency per se changes the gut microbiome, also in co-housed animals [43], which should be taken into account in future studies.

Our study adds adipocytes to the list of cells in which miR-146a fulfills central regulatory functions. Whether it impacts insulin sensitivity mainly at the level of the adipocyte or predominantly via an action on immune cells present in adipose tissue is not clear yet. Both, adipocytes and immune cells express miR-146a, but it was described to be more abundant in the stromal vascular fraction of WAT compared to adipocytes [8]. The miRNA may act in a cell-intrinsic manner but can also be released and exert auto-/paracrine and even endocrine effects. As such, adipose tissue macrophages secrete exosomal miRNAs and transfer them to insulin-sensitive tissues [44]. Another open question is if adipose tissue is the main player in regulating systemic insulin sensitivity and glucose tolerance or if other organs, such as the liver and skeletal muscle, are involved as well. Future cell type-specific knockouts of miR-146a^−/−^ will aid addressing these questions in detail.

## Conclusion

Taken together, our study highlighted miR-146a as a crucial regulator of cellular and systemic insulin sensitivity. We identified NPR3 as novel target gene of miR-146a and discovered that genetic ablation of NPR3 increases insulin sensitivity in adipocytes. MiR-146a and its target genes may, thus, constitute attractive therapeutic targets in the fight of obesity and its associated diseases.

## Materials and Methods

### Animal experiments

All animal studies were approved by the local authorities (Regierungspräsidium Tübingen) and experiments were performed in accordance with Germany’s laws and the rules and regulations governing animal research in the European Union. Female C57BL/6 J and B6.Cg-Mir146atm1.1Bal/J [9, 10] were obtained from the Jackson Laboratory (Bar Harbor, USA). Mice were housed at 22–24 °C with a 14/10 h light/dark cycle and ad libitum access to food and water. At an age of 10 weeks, chow diet was changed to either control diet (normal diet, ND, D12450B mod. LS 13 kJ% fat from vegetable oil) or high-fat diet (HFD, D12492n(I) mod. 60 kJ% fat from lard) (Ssniff Spezialdiäten, Soest, Germany). Body weights were measured twice per week.

### Insulin tolerance test (ITT)

The animals were weighed and basal blood glucose was measured with a standard glucometer (Contour next USB, Bayer, Germany). Mice were injected intraperitoneally with 0.75 IU insulin per kg body weight (Novo Nordisk, Mainz, Germany) and blood glucose was measured after 15, 30, 60, 90, and 120 min. During the experiment, the animals had no access to food. After the experiment, the animals were allowed access to their respective diets.

### Oral glucose tolerance test (OGTT)

The animals were fasted for 6 h, weighed, and basal blood glucose was assessed. A small volume of blood was drawn for fasted insulin analysis in plasma. Mice were gavaged with 2.5 g d-glucose per kg body weight and blood glucose was measured after 15, 30, 60, and 120 min. During the experiment, the animals had no access to food. After the experiment the animals were allowed access to their respective diets.

### Organ preparation

After 10 weeks on the respective diets, blood was sampled from the submandibular vein and animals were sacrificed by cervical dislocation. Liver, inguinal (iWAT) and gonadal WAT (gWAT), and spleen were collected and either used freshly for cell isolation, snap-frozen in liquid nitrogen, or fixed in formalin.

### Spleen cell isolation and flow cytometry

Splenocyte isolation, staining of surface molecules and flow cytometry were performed as described earlier [45]. Measurements were performed on a LSR II flow cytometer (BD, Heidelberg, Germany) with antibodies given in Supplemental Table 5. 7-AAD (7-amino-actinomycin-D, Sigma-Aldrich, Taufkirchen, Germany) was used to discriminate living and dead cells.

### Histology

Tissues were fixed overnight in 4% formalin and dehydrated in an ascending alcohol series with 70% (v/v), 95% (v/v), and 100% ethanol, cleared with 100% xylol in a Tissue-Tek RX 11A Rotary Tissue Processor, and then embedded in paraffin. Tissue sections of 10 µm were H&E stained and observed by a certified pathologist.

### Liver triglyceride content

Quantitative liver triglyceride determination was performed as described by Hesse et al*.* with the TR 210 Triglycerides Assay Kit (Randox, Wülfrath, Germany) [46]. Protein concentration of the lysate was determined as described and triglycerides concentration was related to protein concentration (µg triglycerides/µg protein).

### Insulin ELISA

Insulin was measured in plasma samples of fasted mice with the Mouse Ultrasensitive Insulin ELISA (Alpco, Salem, USA). The Homeostatic Model Assessment for Insulin Resistance (HOMA-IR) was calculated according to Parks et al. [47].

### Cell culture

Simpson–Golabi–Behmel syndrome (SGBS) cells were cultured and adipogenic differentiation was induced for 14 days as described previously [12, 13]. Cell culture dishes and disposables were obtained from Sarstedt (Nümbrecht, Germany) and standard chemicals from Sigma-Aldrich. Cell culture media, buffers, and supplements were obtained from ThermoFisher Scientific.

### Transfection, overexpression and knockout studies

Transfection of SGBS adipocytes was performed with Lipofectamine 2000 (ThermoFisher Scientific) as described previously [6]. For miRNA experiments, 20 nM AllStars negative control siRNA, 50 nM miScript inhibitor negative control, 20 nM syn-hsa-miR-146a-5p miScript miRNA mimic, and 50 nM anti-hsa-miR-146a-5p miScript miRNA inhibitor were used (all from Qiagen, Hilden, Germany).

For IRAK1 knockdown experiments shown in the Supplemental Appendix 20 nM siGENOME Human IRAK1 SMARTpool siRNA (M-004760-03-0005) and siGENOME NON-Targeting siRNA Pool #2 (D-001206-14-05) (Dharmacon) were used.

The miR-146a sequence along with its flanking genomic region was first cloned into the pcDNA6.2 EmGFP-miR-neg plasmid and then transferred to the pLenti6.3/V5-DEST plasmid by Gateway recombination (ThermoFisher Scientific). Lentivirus was produced in HEK293FT cells with the help of the psPAX2 and pMD2.G plasmids (gifts from Didier Trono, Addgene #12260 and #12259). SGBS cells in the preadipocyte state were transduced at a MOI of 1 and selected with blasticidin.

SGBS NPR3 knockout cells were generated using CRISPR/Cas9 as described previously [48] using the following sgRNAs: non-targeting sgRNA: 5′ GGT CAC CGA TCG AGA GCT AG 3′, NPR3-sgRNA: 5′ TCC AGA CAG TCA CTC TAC TG 3′.

### Dual Luciferase Reporter Assays

Interaction of miR-146a-5p with two predicted binding sites in the NPR3 mRNAs was assessed using the pmirGLO Dual Luciferase miRNA target expression vector (Promega). Potential binding sites were annotated by miRWalk 3.0 [15] in the 3′ UTR on the transcript NM_001204376 of NPR3 from base pairs 3606–3636 (site 1) and 3782–3805 (site 2). Both binding sites were cloned into the 3′UTR of the firefly luciferase reporter gene encoded on pmirGLO. Mutagenesis of the base pairs complementary to the seed sequence of miR-146a was performed with QuickChange Multi Site-Directed Mutagenesis Kit (Agilent Technologies) with the following primers: site 1: 5′ TTA GGA ACG TAA ATC CCC AAA TAT AAT CAT GAT CAG CAC TAA TCC AGG GTA ACC ATG GGC CAG GCA CCT ACA TTT 3′; site 2: 5′ CTG AAG TTT GAC TGG ATG AGA AAA TAC TGA TAA ATT ATC CTT TTC AGA CTT CTA ACA CCC TGG ATT TCA GGG TAA AAT CCT ATA GAA TTA CTT AAG A 3′. Successful mutagenesis was validated by Sanger sequencing (Eurofins Genomics). For dual luciferase assays, 25 ng of a dual luciferase vector harboring the predicted binding sites of miR-146a-5p and 100 nM of miR-146a-5p mimic or NT siRNA were co-transfected into HEK293 cells using Lipofectamine 2000 (ThermoFisher Scientific) for 48 h. Luciferase activity was quantified using the Dual-Glo Luciferase Assay System (Promega) in a microplate reader (Tecan).

### In vitro glucose uptake

Adipocytes were incubated for 24 h in serum-free medium. Cells were washed once and then incubated for 3 h with HEPES-buffered Krebs–Ringer solution (KRH) completed with 0.05% (m/v) BSA. Cells were then stimulated with increasing doses of insulin as indicated. After 15 min, the cells were either harvested for protein extraction and analysis of Akt phosphorylation at Ser473 or the glucose uptake assay was continued. A glucose working solution composed of KRH with 0.05% (m/v) BSA, 4 µCi/ml ^14^C-deoxy glucose, and 2 mM deoxy glucose was added to final concentrations of 0.2 µCI/ml ^14^C-deoxy glucose and 100 µM deoxy glucose. After additional 15 min, the cells were harvested and the amount of glucose taken up by the cells was measured by liquid scintillation counting on a β-counter.

### In vitro lipogenesis

Adipocytes were incubated for 24 h in serum-free medium. Cells were then stimulated with increasing doses of insulin as indicated and a glucose working solution composed of serum-free media and 10 µCi/ml ^14^C- glucose was added to a final concentration of 0.1 µCI/ml ^14^C- glucose. After 24 h, the cells were harvested, and the amount of glucose taken up by the cells was measured by liquid scintillation counting on a β-counter.

### RNA isolation, reverse transcription and quantitative PCR (qPCR)

Whole tissues were minced in liquid nitrogen. Total RNA isolation was performed with the Direct-zol RNA mini Prep Kit (Zymo Research, Freiburg, Deutschland). For miRNA quantification, total RNA was reverse-transcribed using the miScript II RT Kit (Qiagen). For mRNA quantification, total RNA was reverse-transcribed using SuperScript II Reverse Transcriptase (ThermoFisher Scientific). mRNA and miRNA expression were quantified on a CFX Connect Real-Time PCR Detection System (BioRad, München, Germany). As reference genes, sno68 (miRNA) and HPRT (mRNA) were used. Primers used are given in Supplemental Tables 2, 3, and 4.

### Microarray analyses

Microarray analyses were performed using 200 ng total RNA as starting material and 5.5 μg ssDNA per hybridization (GeneChip Fluidics Station 450; Affymetrix, Santa Clara, CA). Total RNAs were amplified and labeled following the Whole Transcript Sense Target Labeling Assay (http://www.affymetrix.com). Labeled ssDNA was hybridized to Mouse Gene 2.0 ST Affymetrix GeneChip arrays. (Affymetrix, Santa Clara, CA). The chips were scanned with an Affymetrix GeneChip Scanner 3000 and subsequent images analyzed using Affymetrix Expression ConsoleTM Software (Affymetrix). Transcriptome analyses were performed using BRB-ArrayTools developed by Dr. Richard Simon and BRB-ArrayTools Development Team (http://linus.nci.nih.gov/BRB-ArrayTools.html). Raw feature data were normalized and log 2 intensity expression summary values for each probe set were calculated using robust multiarray average [49]. Filtering: Genes showing minimal variation across the set of arrays were excluded from the analysis. Genes whose expression differed by at least 1.5-fold from the median in at least 20% of the arrays were retained. Class comparison: Genes were identified as differentially expressed among the two classes using a 2 sample t-test. Genes were considered statistically significant if their p-value was less than 0.05 and displayed a fold change between the two groups of at least 1.5-fold. Benjamini and Hochberg correction was used to calculate the false discovery rate [50]. Complete microarray data are available at Gene Expression Omnibus (GEO accession number: GSE151268).

### Western Blot

Western Blot was performed as described previously [6]. Following antibodies were used: anti-NPR3 (EPR12716, Abcam, Berlin, Germany), anti-pAKT (Ser473; #9271, Cell Signaling, Frankfurt, Germany), anti-AKT (#9272, Cell Signaling), anti-adiponectin (#GTX112777, GeneTex, Taiwan), anti-IRAK1 (SC-5287, Santa Cruz, Heidelberg, Germany), anti-IRAK1 (#4504, Cell Signaling), anti-TRAF6 (PA29622, Invitrogen, Carlsbad, USA), anti-α-tubulin (DM1A, Merck, Darmstadt, Germany), anti-β-actin (AC-15, Sigma-Aldrich), anti-GAPDH (6C5, HyTest, Turku, Finland), hFAB rhodamine anti-GAPDH, anti-tubulin, and anti-actin (#12004168, #12004166, #12004164, BioRad, Feldkirchen, Germany).

### Statistics

GraphPad Prism (version 7.01, GraphPad Software, La Jolla, USA) software was used to perform statistical analyses. For comparisons between two groups, the student’s *t* test was used, whereas for three or more groups, the one-way ANOVA (for one independent variable) or the two-way ANOVA (for two-independent variables) was used. Multiple comparisons were corrected by the Tukey or Bonferroni methods. Statistical significance was assumed for *p* values < 0.05.

### Electronic supplementary material

Below is the link to the electronic supplementary material.

Supplementary file1 (PDF 1642 KB)
