# Supplementary material for: miR-146a regulates insulin sensitivity via NPR3
Source: Cell Mol Life Sci. 2020 Nov 18;78(6):2987–3003. doi: 10.1007/s00018-020-03699-1 (PMC8004521; doi:10.1007/s00018-020-03699-1)
Supplement: Supplementary file 1 — Supplementary file1 (PDF 1642 KB) [file 18_2020_3699_MOESM1_ESM.pdf]

Supplemental Appendix

miR-146a regulates insulin sensitivity *via* NPR3

Roos J [1,2], Dahlhaus M [1,2], Funcke JB [1,2,3], Kustermann M [1], Strauss G [1], Halbgebauer D [1,2], Boldrin E [1], Holzmann K [4], Möller P [5], Trojanowski BM [6], Baumann B [6], Debatin KM [1], Wabitsch M [1,2], Fischer-Posovszky P [1,2]

Short title: miR-146a and insulin sensitivity

Affiliations:
[1] Department of Pediatrics and Adolescent Medicine, University Medical Center, Ulm, Germany; [2] Division of Pediatric Endocrinology and Diabetes, Department of Pediatrics and Adolescent Medicine, University Medical Center, Ulm, Germany; [3] Touchstone Diabetes Center, Department of Internal Medicine, The University of Texas Southwestern Medical Center, Dallas, Texas, USA; [4] Core Facility Genomics, Ulm University, Ulm, Germany; [5] Institute of Pathology, University Medical Center, Ulm, Germany; [6] Institute of Physiological Chemistry, Ulm University, Ulm, Germany;

Corresponding author:
Prof. Dr. Pamela Fischer-Posovszky
Orcid: 0000-0003-3402-9840
Department of Pediatrics and Adolescent Medicine
University Medical Center Ulm
Eythstr. 24
89075 Ulm
Germany
Tel: +49 731 500 57425
Fax: +49 731 500 57246
Email: pamela.fischer@uniklinik-ulm.de

Supplemental Figures

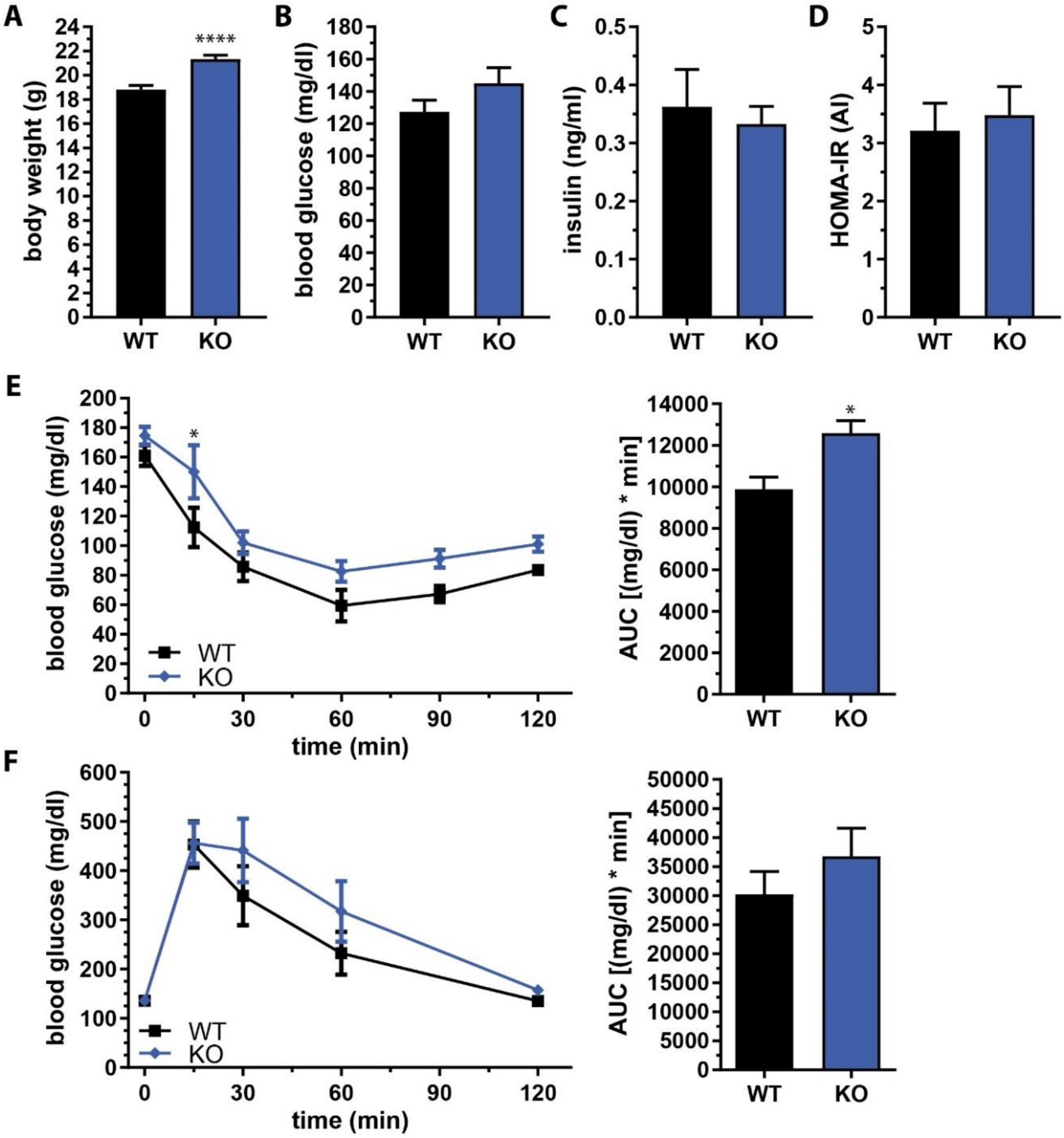

Figure S1 | Basal metabolic characteristics of miR-146a$^{-/-}$ mice. Female miR-146a$^{-/-}$ (KO) and respective control mice (WT) were studied at an age of 10 weeks. **(A)** Body weight, **(B)** fasted blood glucose, **(C)** fasted plasma insulin concentrations, and **(D)** HOMA-IR. **(E)** To assess insulin sensitivity, mice were intraperitoneally injected with 0.75 IU insulin per kg body weight and blood glucose was monitored for 120 min (ITT). Area under the curve (AUC) of ITT. **(F)** To assess glucose tolerance, mice were gavaged with 2.5 g glucose per kg body weight and blood glucose was monitored for 120 min (OGTT). AUC of OGTT. Data are displayed as mean and SEM of 5 animals per group. Statistics: (A-D, E and F AUCs) unpaired t-test, (E and F curves) two-way ANOVA with Bonferroni correction. * $p<0.05$, **** $p<0.0001$.

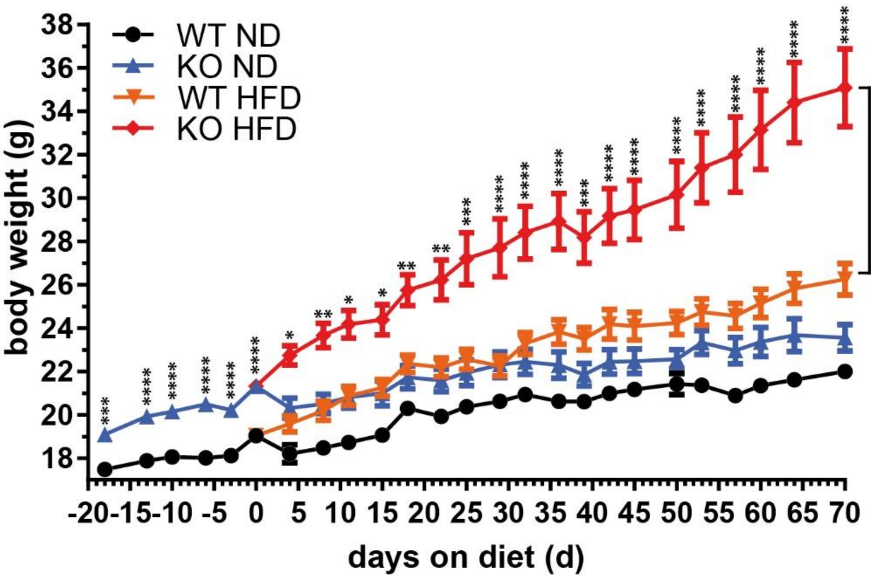

Figure S2 | Body weight development. Female miR-146a$^{-/-}$ (KO) and respective control mice (WT) at an age of 10 weeks were fed a high fat diet (HFD) or normal diet (ND). Body weight was measured twice a week. Data are displayed as mean and SEM of 10 animals per group. Statistics: two-way ANOVA with Bonferroni correction. * p<0.05, * p<0.01, * p<0.001, * p<0.0001.

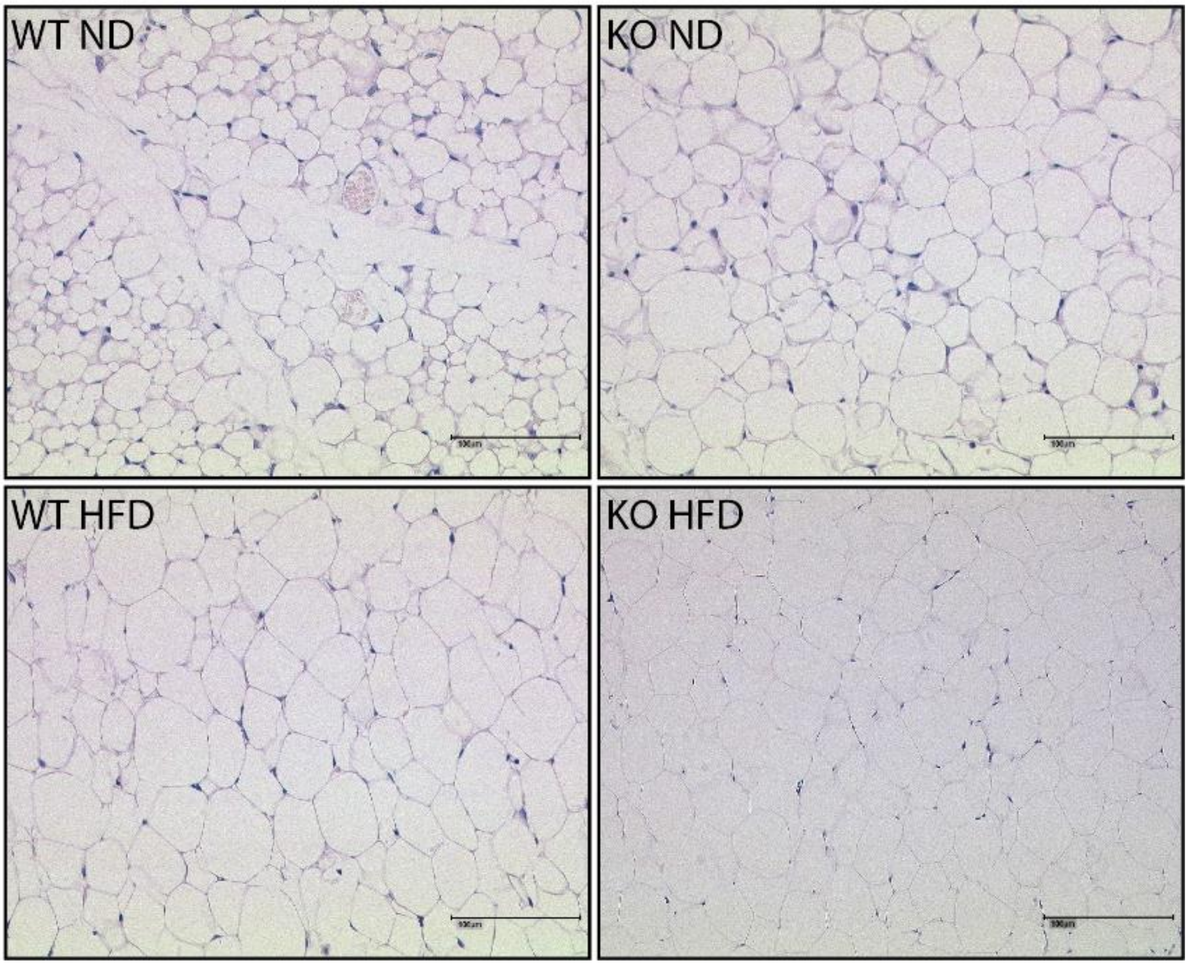

Figure S3 | Inguinal fat pad histology. After 10 weeks of high fat diet (HFD) or respective normal diet (ND), inguinal white adipose tissue (iWAT) of miR-146a$^{-/-}$ (KO) or respective control mice (WT) was collected and processed for histological analysis. Shown are representative microphotographs of H&E stained iWAT sections.

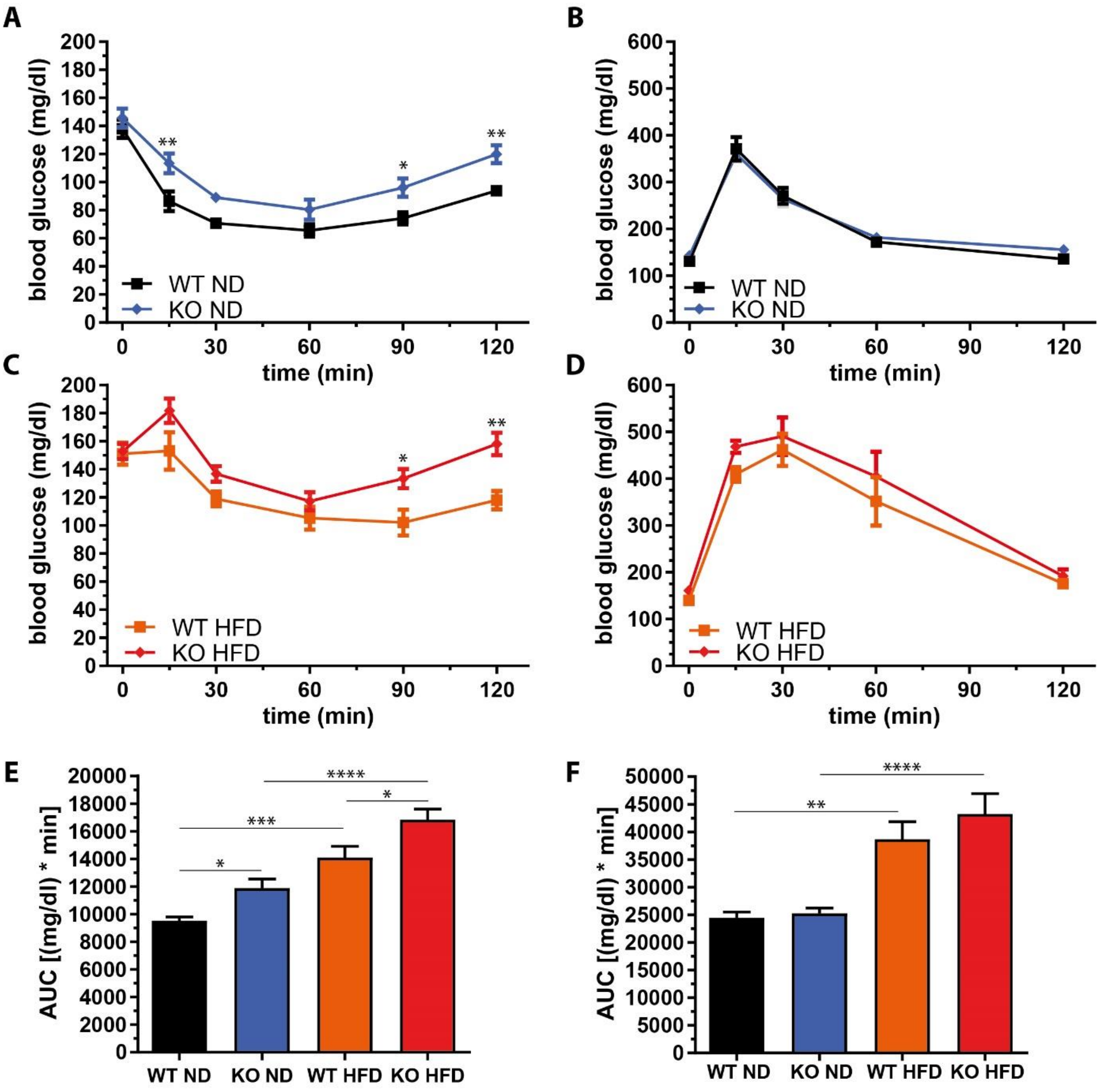

Figure S4 | Metabolic characterisation of miR-146a$^{-/-}$ mice after 5 weeks on a high fat diet. Female miR-146a$^{-/-}$ (KO) and respective control mice (WT) were metabolically characterized at the age of 15 weeks, after 5 weeks high fat (HFD) or respective normal diet (ND). **(A and B)** Insulin tolerance test (ITT) and oral glucose tolerance test (OGTT) in mice fed a ND. **(C and D)** ITT and OGTT in mice fed an HFD. **(E)** Area under the curve (AUC) of ITT. **(F)** AUC of OGTT. Data are displayed as mean and SEM of 10 animals per group. Statistics: (A-D) two-way ANOVA with Bonferroni correction. (E and F) one-way ANOVA with Tukey correction. * $p<0.05$, ** $p<0.01$, *** $p<0.001$, **** $p<0.0001$.

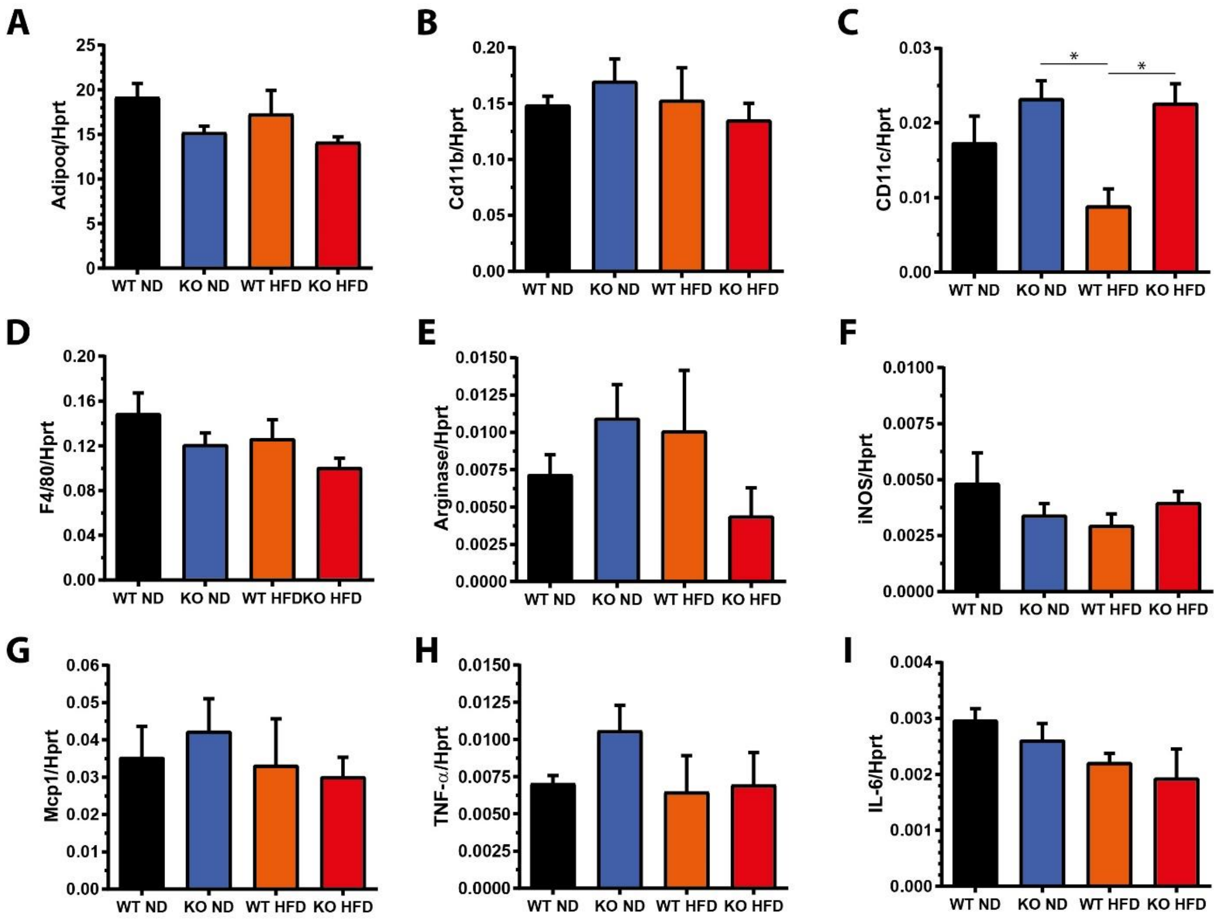

Figure S5 | Markers for infiltrating immune cells in inguinal adipose tissue. After 10 weeks of high fat (HFD) or normal diet (ND) miR-146a$^{-/-}$ (KO) or respective control mice (WT) were sacrificed, inguinal white adipose tissue (iWAT) was collected and processed for qPCR. mRNA expression is given in relation to Hprt as reference gene ($2^{-\Delta CT}$). **(A)** Adiponectin (Adipoq), **(B)** cluster of differentiation 11b (Cd11b), **(C)** cluster of differentiation 11c (Cd11c), **(D)** EGF-like module-containing mucin-like hormone receptor-like 1 (F4/80), **(E)** arginase, **(F)** iNOS, **(G)** monocyte chemoattractant protein 1 (Mcp-1), **(H)** tumor necrosis factor α (Tnf-α), and **(I)** interleukin 6 (IL-6) expression. Data are displayed as mean and SEM of 5 animals per group. Statistics: One-way ANOVA with Tukey correction. * $p < 0.05$, ** $p < 0.01$.

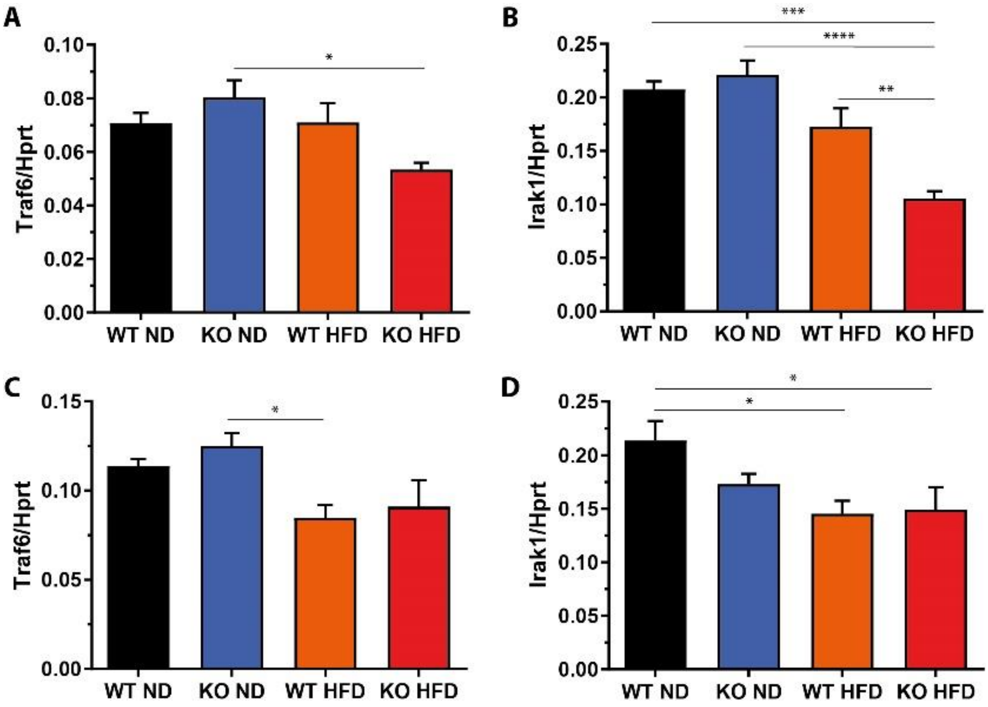

Figure S6 | Irak1 and Traf6 mRNA expression in gonadal and inguinal fad pads.

After 10 weeks of high fat diet (HFD) or respective normal diet (ND) gonadal (gWAT) and inguinal white adipose tissue (iWAT) of miR-146a$^{-/-}$ (KO) or respective control mice (WT) were collected and processed for qPCR. RNA expression is given in relation to Hprt as the reference gene ($2^{-\Delta CT}$). **(A)** gWAT Traf6 and **(B)** Irak1. **(C)** iWAT Traf6 and **(D)** Irak1**.** Data are displayed as mean and SEM of 5 animals per group. Statistics: one-way ANOVA with Tukey correction. * $p<0.05$, ** $p<0.01$, *** $p<0.001$, **** $p<0.0001$.

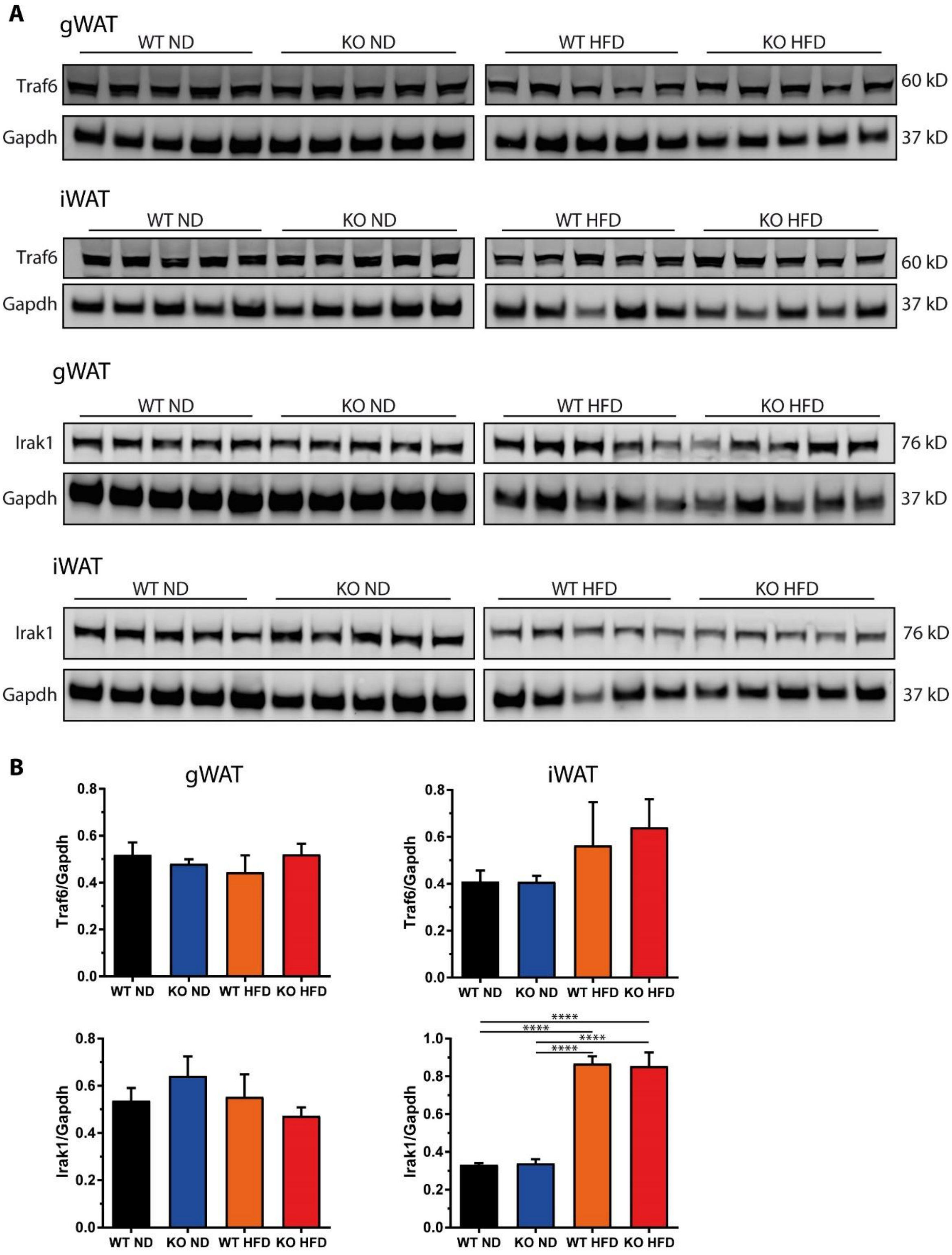

Figure S7 | Irak1 and Traf6 protein expression in murine WAT. (A) Irak1 and Traf6 protein expression in inguinal and gonadal white adipose tissue (iWAT, gWAT) of control mice (WT) or miR-146a$^{-/-}$ mice (KO) after 10 weeks of normal (ND) or high fat diet (HFD) with **(B)** densitometric analysis of 5 animals per group displayed as mean and SEM. Statistics: one-way ANOVA with Tukey correction, **** p<0.0001.

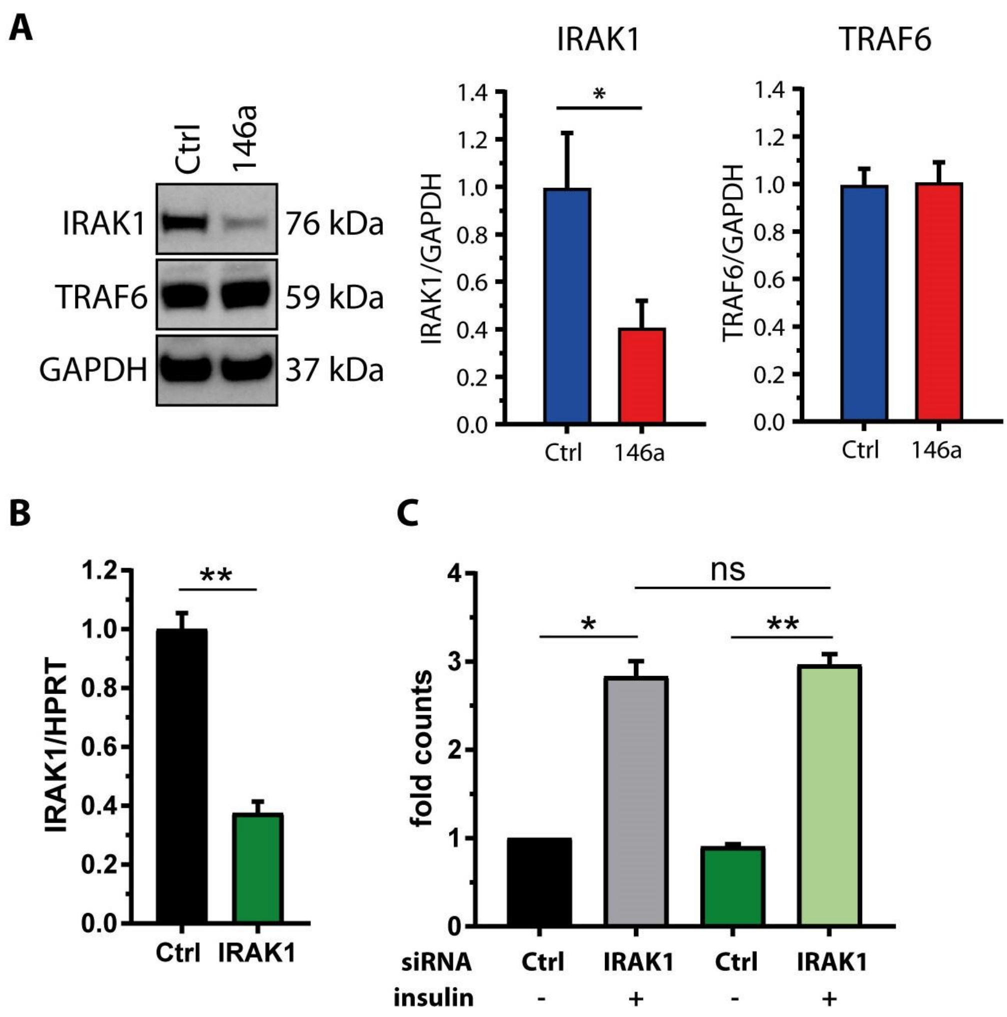

Figure S8 | IRAK1 is regulated by miR-146a in SGBS adipocytes but does not regulate insulin stimulated glucose uptake (A) Representative IRAK1 and TRAF6 Western blot of SGBS adipocytes overexpressing miR-146a with densitometric analysis of 3 independent performed experiments displayed as mean and SEM. SGBS adipocytes were transfected with 20 nM control (Ctrl) or IRAK1 siRNA and **(B)** IRAK1 expression was controlled by qPCR and **(C)** glucose uptake experiments were performed with 0 nM and 1 nM insulin. Data are displayed as mean and SEM fold increase to Ctrl siRNA 0 nM insulin. Statistics: (A,B) paired t-test, * $p<0.05$; (C) one-way ANOVA with Tukey correction, ns = not significant, * $p<0.05$, ** $p<0.01$.

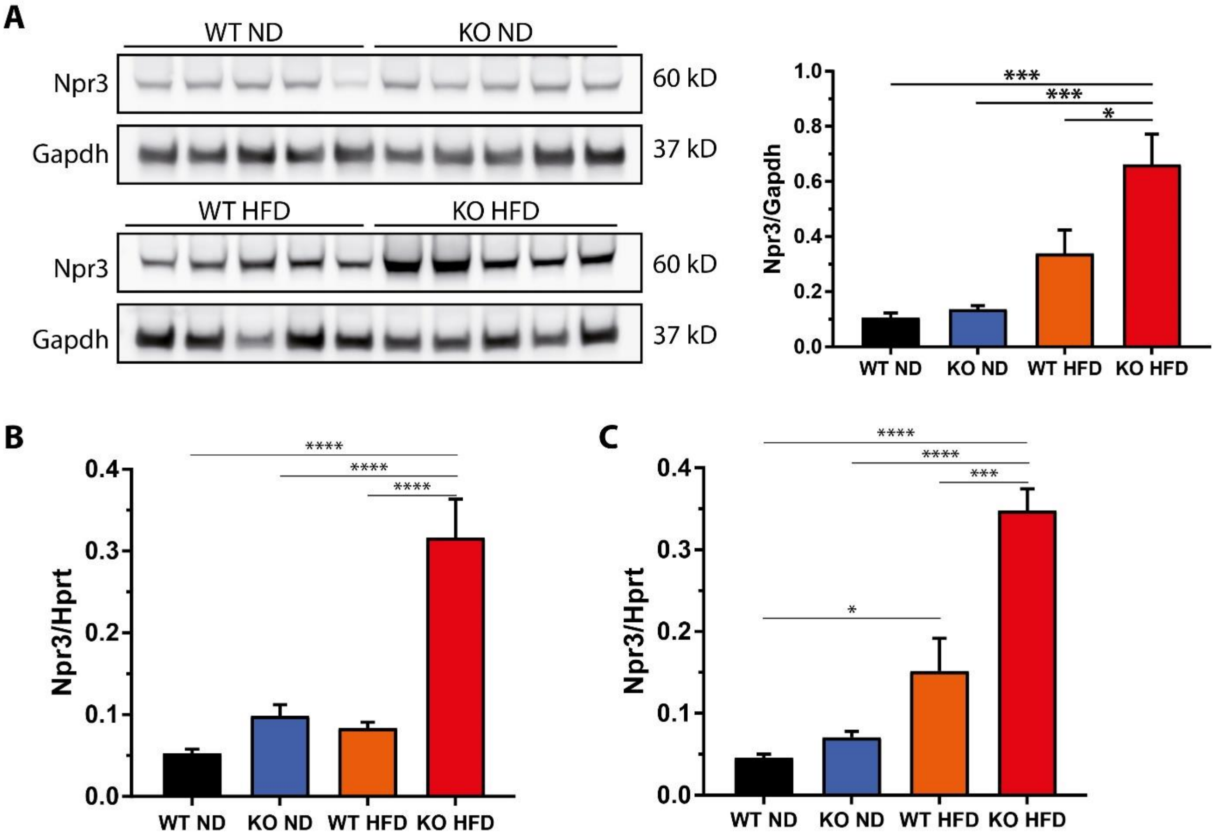

Figure S9 | NPR3 is a target gene of miR-146a in murine WAT. (A) Npr3 protein expression in inguinal white adipose tissue (iWAT) of control mice (WT) or miR-146a$^{-/-}$ mice (KO) after 10 weeks of normal (ND) or high fat diet (HFD) with densitometric analysis of 5 animals per group displayed as mean and SEM. **(B)** NPR3 mRNA expression in gonadal WAT (gWAT) and **(C)** iWAT. Statistics: one-way ANOVA with Tukey correction, * p<0.05, *** p<0.001, **** p<0.0001.

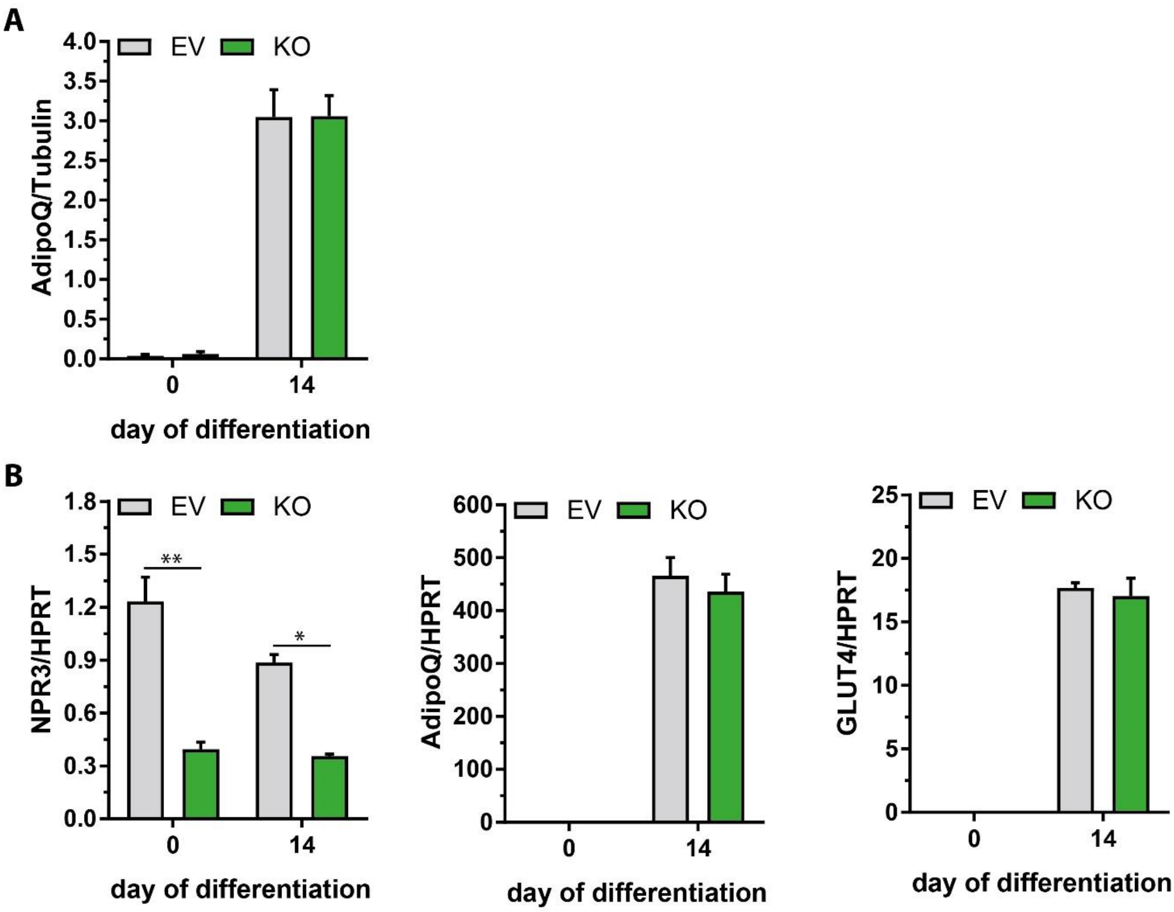

Figure S10 | NPR3 ablation does not affect adipogenic differentiation. (A) Densitometric analysis for adiponectin Western Blots in pre-adipocytes (d0) and adipocytes (d14) displayed as mean and SEM of 4 independent replicates. **(B)** NPR3, adiponectin (AdipoQ), and glucose transporter 4 (GLUT4) mRNA expression in pre-adipocytes and adipocytes of control (EV) and NPR3 KO cells (KO). Data are displayed as mean and SEM of 4 independent experiments. Statistics: one-way ANOVA with Tukey correction, * $p<0.05$, ** $p<0.01$.

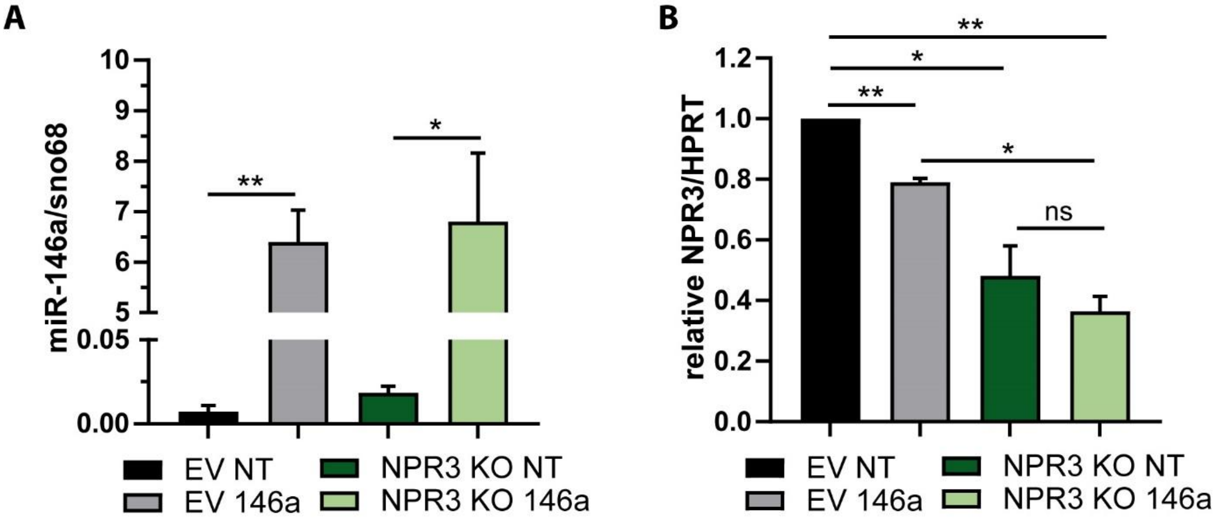

Figure S11 | miR-146a and NPR3 mRNA levels of NPR3 KO cells after NT and control (NT) and miR-146a mimic transfection. Control (EV) or NPR3 KO cells were transfected with 20 nM non-targeting (NT) or miR-146a mimic and **(A)** miR-146a expression and **(B)** NPR3 mRNA expression was quantified by qPCR in mature adipocytes. Data are displayed as mean and SEM of 3 independent experiments with sno68 or HPRT as reference gene. Statistics: one-way ANOVA with Tukey correction, ns= not significant, * $p<0.05$, ** $p<0.01$.

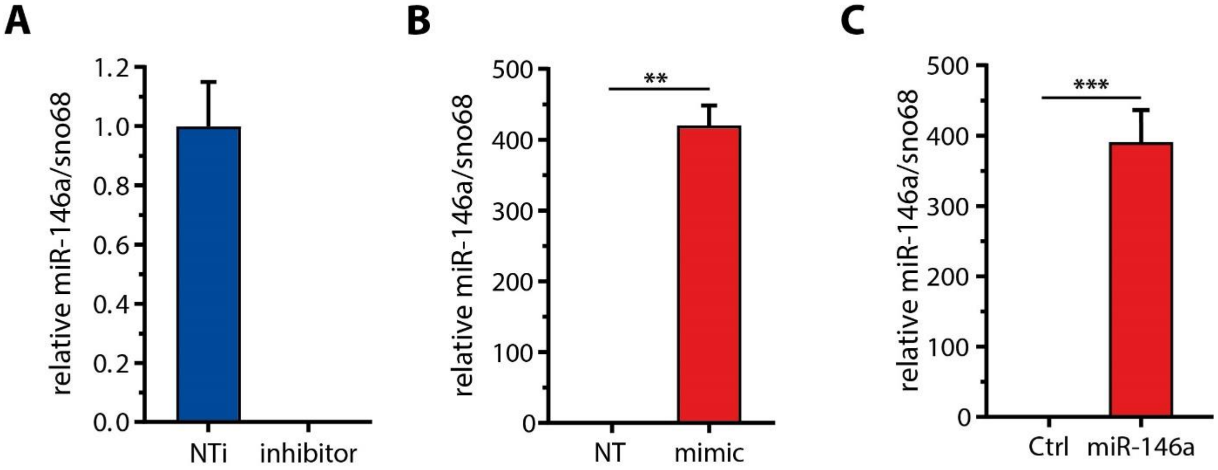

Figure S12 | miR-146a levels. Transfection efficiency and miR-146a overexpression were measured by qPCR and given as $2^{-\Delta\Delta CT}$ with sno68 as the reference gene. **(A)** miR-146a inhibitor (50 nm) or inhibitor control (NTi, 50 nM) transfected adipocytes. **(B)** miR-146a mimic (20 nM) or control (NT, 20 nM) transfected adipocytes. **(C)** Adipocytes stably overexpressing miR-146a or non-target control (Ctrl). Data are displayed as mean and SEM of 4 (A) or 3 (B and C) independent experiments. Statistics: paired t-test, * p<0.05, ** p<0.01.

Supplemental Table 1 | Number of differentially expressed genes

	WT ND	*WT HFD*	*KO ND*	*KO HFD*
WT ND	*X*	*458*	*68*	*/*
WT HFD	*458*	*X*	*/*	*516*
KO ND	*68*	*/*	*X*	*748*
KO HFD	*/*	*516*	*748*	*X*

Supplemental Table 2 | Murine qPCR primer pairs

target gene	primer	sequence 5’>3’
**Adipoq**	forward	GTTCCTCTTAATCCTGCCCAGTCATGCC
	reverse	GGACCAAGAAGACCTGCATCTCCTTTCTC
**Arginase**	forward	TCCTTTCAAATTGTGAAGAACCCACGGTC
	reverse	AGAATCCTGGTACATCTGGGAACTTTCCT
**Cd11b**	forward	AGGCTCTCAGAGAATGTCCTCAG
	reverse	TCCATCACAGTTGAGACAAACTCC
**Cd11c**	forward	CAGTCTGGCAGATGTGGCTA
	reverse	GGATCTGGGATGCTGAAATC
**F4/80**	forward	CTTTGGCTATGGGCTTCCAGTC
	reverse	GCAAGGAGGACAGAGTTTATCGTG
**Glut4**	forward	TGAGCTGAAGGATGAGAAACGGAAGTTGGA
	reverse	CTAAGAGCACCGAGACCAACGTGAAGACC
**I-Ab**	forward	GGTGTGAGTCCTGGTGACTG
	reverse	GTACACGAAATGCCTTTCGGAG
**Il-6**	forward	GATGGATGCTACCAAACTGGA
	reverse	TCTGAAGGACTCTGGCTTTG
**iNos**	forward	AGCAATGGGCAGACTCTGAAGAAATCTC
	reverse	ATGTTTGCTTCGGACATCAAAGGTCTCAC
**Npr3**	forward	GGTGGCCTACGAAGACTCG
	reverse	CCAGGATGAGATCCGGCTTG
**Mcp-1**	forward	AGGTCCCTGTCATGCTTCTG
	reverse	GGGATCATCTTGCTGGTGAA
**Mpo**	forward	AGTTGTGCTGAGCTGTATGGA
	reverse	GCTCCGCTTGATGCTTTCTC
**SiglecF**	forward	GCTCAGTGTCATCTATGCTCCA
	reverse	CATACAGACCAGGCTCAGGGA
**Tnf-α**	forward	CCAGACCCTCACACTCAGATCATCTTCTC
	reverse	CTAGTTGGTTGTCTTTGAGATCCATGCCGT

Supplemental Table 3 | QIAGEN miScript Primer assays

primer assay	target ncRNA	category number
**Hs-SNORD68_11**	SNO68 snoRNA	MS00033712
**Hs-miR-146a_1**	hsa-miR-146a-5p	MS00003535
**Mm_miR-146_1**	mmu-miR-145a-5p	MS00001638

Supplemental Table 4 | Human qPCR primer pairs

target gene	primer	sequence 5'>3'
**AdipoQ**	forward	GGCCGTGATGGCAGAGAT
	reverse	CCTTCAGCCCCGGGTACT
**FOLH1**	forward	ACACAGATACCACATTTAGCAGG
	reverse	TTTGGGTAGGACAACAGGACA
**GLUT4**	forward	TTCCAACAGATAGGCTCCGAAG
	reverse	AAGCACCGCAGAGAACACAG
**MEST**	forward	AGTTGTGCTTTTACACGGTTTTC
	reverse	CAAGGGCAATCACCCGATGAA
**NPR3**	forward	ACAGCAGACTTGGAACAGAAC
	reverse	AATCCCCATATCGGTCTCCGT
**SLC4A1**	forward	GGTGATGGACGAAAAGAACCA
	reverse	AAGACTCTACGCAGCTCTAGG

SupplementalTable 5 | Antibodies for flow cytometry

epitope	clone	fluorochrome	company	dilution
**CD11b**	M1/70	APC eFl780	eBioscience	1:150
**CD11c**	N418	PE-Cy7	ThermoFisher Scientific	1:1000
**CD19**	1D3	PE	BD	1:600
**CD3**	17A2	AF700	ThermoFisher Scientific	1:40
**CD4**	RM4-5	APC-eFl780	ThermoFisher Scientific	1:300
**CD45**	30-F11	AF700	BioLegend	1:400
**CD8**	53-6.7	PE-Cy7	ThermoFisher Scientific	1:1500
**F4/80**	BM6	FITC	ThermoFisher Scientific	1:50
**Gr-1**	RB6-8C5	eFl450	ThermoFisher Scientific	1:100
**I-Ab**	M5/114.15.2	APC	ThermoFisher Scientific	1:2000
**NK1.1**	PK136	PE	ThermoFisher Scientific	1:50
**SiglecF**	E50-2440	PE	BD	1:50
**TCRβ**	H57-597	APC	ThermoFisher Scientific	1:200
